# Immunogenicity of an adjuvanted broadly active influenza vaccine in immunocompromised and diverse populations

**DOI:** 10.1002/btm2.10634

**Published:** 2023-12-08

**Authors:** Dylan A. Hendy, Erik S. Pena, Luis Ontiveros‐Padilla, Timothy A. Dixon, Denzel D. Middleton, Grace L. Williamson, Nicole Rose Lukesh, Sean R. Simpson, Rebeca T. Stiepel, Md Jahirul Islam, Michael A. Carlock, Ted M. Ross, Eric M. Bachelder, Kristy M. Ainslie

**Affiliations:** ^1^ Division of Pharmacoengineering and Molecular Pharmaceutics, Eshelman School of Pharmacy University of North Carolina at Chapel Hill Chapel Hill North Carolina USA; ^2^ Joint Department of Biomedical Engineering University of North Carolina at Chapel Hill and North Carolina State University Chapel Hill North Carolina USA; ^3^ Florida Research and Innovation Center Port St. Lucie Florida USA; ^4^ Center for Vaccines and Immunology University of Georgia Athens Georgia USA; ^5^ Department of Infectious Diseases University of Georgia Athens Georgia USA; ^6^ Department of Microbiology and Immunology, UNC School of Medicine University of North Carolina Chapel Hill North Carolina USA

**Keywords:** aging, chemotherapy, collaborative cross, obesity, polymeric microparticles

## Abstract

Influenza virus outbreaks are a major burden worldwide each year. Current vaccination strategies are inadequate due to antigenic drift/shift of the virus and the elicitation of low immune responses. The use of computationally optimized broadly reactive antigen (COBRA) hemagglutinin (HA) immunogens subvert the constantly mutating viruses; however, they are poorly immunogenic on their own. To increase the immunogenicity of subunit vaccines such as this, adjuvants can be delivered with the vaccine. For example, agonists of the stimulator of interferon genes (STING) have proven efficacy as vaccine adjuvants. However, their use in high‐risk populations most vulnerable to influenza virus infection has not been closely examined. Here, we utilize a vaccine platform consisting of acetalated dextran microparticles loaded with COBRA HA and the STING agonist cyclic GMP‐AMP. We examine the immunogenicity of this platform in mouse models of obesity, aging, and chemotherapy‐induced immunosuppression. Further, we examine vaccine efficacy in collaborative cross mice, a genetically diverse population that mimics human genetic heterogeneity. Overall, this vaccine platform had variable efficacy in these populations supporting work to better tailor adjuvants to specific populations.

**Translational Impact Statement**

Our acetalated dextran (Ace‐DEX) microparticle (MP) platform has illustrated enhanced delivery of the stimulator of interferon genes (STING) agonist cyclic GMP‐AMP (cGAMP) over other particle systems as well as generation of long‐term protective immunity in mice and ferrets. Here, we present the unique efficacy of a broadly active influenza vaccine adjuvanted with cGAMP MPs in mouse models for three different populations that are highly susceptible to influenza (obesity, aging, and chemotherapy‐induced immunosuppression) and one genetically diverse population (the collaborative cross). We show that the vaccine platform demonstrated variable efficacy across these different models, which is a key finding to inform future development of STING agonist‐based vaccines for infectious disease and cancer.

## 1 INTRODUCTION

In the United States, influenza is estimated to cause up to 41 million illnesses, 710,000 hospitalizations, and 52,000 deaths annually.^1^ The impact of the disease is largely due to inadequacies with current seasonal vaccination strategies. Seasonal vaccines rely on generating immunity against the immunodominant hemagglutinin (HA) protein that changes every year due to antigenic drift and shift.^2^ This means scientists must redesign yearly the vaccine to incorporate emerging strains of influenza. The ability to predict which strain will be circulating each year is not perfect, which leads to seasonal vaccines with low efficacy.^3^ Even if there is a perfect match, current inactivated influenza vaccines have limited immunogenicity in immunocompromised populations. For example, elderly, obese, and individuals on immunosuppressive drug regimes, have all shown to have decreased response to inactivated influenza vaccines compared to healthy individuals.^4^, ^5^, ^6^, ^7^, ^8^ To improve vaccine response in these populations, the FDA approved high dose inactivated influenza vaccine (high‐dose Fluzone; 2009; Sanofi Pasteur Inc.) and an adjuvanted inactivated influenza vaccine (FluAd; 2015; CSL Seqirus). Both vaccines offer improved immunogenicity in elderly populations but still exhibit limitations. Both produce a potent Th2 response but not a strong Th1 response,^9^, ^10^ and the generation of a Th1 immune response has been shown to be important in protecting immunocompromised populations.^11^ Given these inadequacies with current seasonal inactivated influenza vaccines, much research has been done to improve them.

First, to subvert the mutating virus, work has been done to identify antigens that are conserved among virus strains and clades (i.e. universal antigens). Two examples of such antigens are the matrix‐2 protein ectodomain (M2e) and the stalk region of HA stalk.^12^, ^13^ While these antigens are highly conserved season to season, there have been some barriers to their clinical translation. For example, both antigens generate a poor neutralizing antibody response.^14^ Further, antibodies generated by HA stalk vaccines have been associated with autoreactivity and enhanced viral pathogenesis.^15^, ^16^ Computationally optimized broadly reactive antigen (COBRA) HA is an alternative to these two conserved antigens. COBRA is the product of a methodology to design broadly active HA antigens that have been shown to generate protective immunity against past, present, and future strains of influenza. An additional advantage of COBRA HA over other antigens is that COBRA produces a strong neutralizing antibody response.^17^ However, on its own, COBRA HA is not very immunogenic and needs to be paired with an adjuvant.

Cyclic GMP‐AMP (cGAMP) is a cyclized dinucleotide and agonist of the stimulator of interferon genes (STING) pathway. STING activation induces the production of inflammatory type I interferons, which strongly facilitate the development of Th1‐dominanted immune responses.^18^ While cGAMP is a potent STING activator, its efficacy is limited by its extreme hydrophilicity and inability to freely cross the cell membrane. Our group has extensively researched acetalated dextran (Ace‐DEX) microparticles (MPs) as a carrier for vaccine adjuvants such as cGAMP to enhance cell delivery. Ace‐DEX MPs possess many advantageous properties for vaccine delivery such as acid sensitivity, tunable degradation, passive targeting to phagocytic cells, and enhanced MHC I cross presentation.^19^ Our group has shown that an influenza subunit vaccine adjuvanted with cGAMP‐loaded Ace‐DEX MPs encapsulates the dinucleotide adjuvant more effectively than poly(lactic‐*co*‐glycolic acid) (PLGA) MPs and stimulates IFN‐γ at significantly higher levels in immune cells than PLGA MPs or liposomes containing cGAMP.^20^ Further, we have shown that cGAMP Ace‐DEX MPs induce a balanced Th1/Th2 response, protection after lethal influenza challenge in mice and ferrets, and a long‐term immune response (1 year) in ferrets.^20^, ^21^, ^22^ The encapsulation of antigens in Ace‐DEX MPs also enhances immunogenicity.^22^, ^23^ We have previously shown that when the model antigen OVA is encapsulated in Ace‐DEX MPs there is enhanced MHC I and MHC II presentation in vitro.^24^ Furthermore, vaccinating mice with M2e loaded Ace‐DEX MPs leads to the expansion of antigen specific CD4+ T‐cells as determined by tetramer staining.^25^

With this previous work in mind, we sought to encapsulate both the COBRA H3 HA immunogen J4 and cGAMP into Ace‐DEX MPs as a broadly active influenza vaccine platform. J4 and cGAMP were encapsulated separately in MPs due to previous work that has shown separately encapsulated antigen and adjuvant are more immunogenic and protective against a lethal challenge than co‐encapsulation.^23^, ^26^, ^27^ Given the potent Th1/Th2 response produced by cGAMP MPs, we hypothesized that this platform would be especially useful in immunocompromised populations such as obese, elderly, and chemotherapy‐induced immunosuppressed individuals where this balanced response has been shown to be important to vaccine immunogenicity.^11^ Further, there have been numerous reports of using STING agonists as vaccine adjuvants, but very few studies investigating their efficacy in immunocompromised populations.^28^ Therefore, J4 and cGAMP MPs were used to vaccinate aged mice, obese mice, and chemotherapy‐induced immunosuppressed mice to investigate the utility of our vaccine platform in these models.

To evaluate the translatability of this platform, it is also important to consider the genetic diversity of the population that will receive the vaccine, and a single mouse model does not sufficiently capture this diversity. In fact, in any given year, an influenza vaccine may only cause 50% of those who receive it to seroconvert, and some of this inconsistency could be explained by genetic diversity.^29^, ^30^ Collaborative cross (CC) mice are a genetic reference population created by crossbreeding several common laboratory mouse strains (Figure S1).^31^ Work examining the susceptibility of CC mice to influenza has demonstrated that many of the differentially expressed genes between influenza‐resistant and ‐susceptible strains are similar to that of humans.^32^, ^33^ To this end, we examined the immunogenicity of our broadly active influenza vaccine platform in CC mice to determine whether our vaccine could be effective in a genetically diverse population. Overall, this work aims to examine the translatability of a cGAMP MP adjuvanted broadly active influenza vaccine by determining its efficacy in many clinically relevant mouse models.

## 2 MATERIALS AND METHODS

### 2.1 Materials

All chemicals were purchased from Sigma (St. Louis, MO) unless otherwise indicated. Assays, biologics, and disposables were purchased from Thermo Fisher Scientific (Waltham, MA) unless otherwise indicated.

### 2.2 COBRA HA immunogen expression

COBRA HA immunogens were expressed by transfecting the truncated COBRA J4 gene cloned into the pcDNA3.1^+^ plasmid into HEK293T suspension cells (Thermo Fisher Scientific) as previously described.^34^

### 2.3 Mice

All procedures involving mice were conducted with approval of the University of North Carolina at Chapel Hill Institutional Animal Care and Use Committee (IACUC).

For the obesity study, 3‐week‐old female C57BL/6J mice were purchased from Jackson Laboratory (Bar Harbor, ME). Mice were given access to food and water ad libitum. Non‐diet induced obese mice (non‐DIO) were fed a standard rodent diet provided by animal husbandry (Lab Diet, 5V0F), and diet induced obese mice (DIO) were fed a Rodent diet containing 60 kcal% fat (Research Diets, D12492). Mice were weighed weekly, and after 16 weeks on the diet, mice over 40 g were enrolled in the vaccination study. Mice remained on the high fat diet for the entirety of the vaccination study.

For the aged study, 10‐month‐old female retired breeder C57BL/6J mice were purchased from Charles River Laboratories (Wilmington, MA). Mice were housed until they reached 18 months old, at which point the vaccination study began as detailed below. Before the vaccination study began, 6‐ to 8‐week‐old female C57BL/6J mice were purchased from Charles River Laboratories to serve as young controls.

For the cyclophosphamide (CP) study, 6‐ to 8‐week‐old female C57BL/6J mice were purchased from Jackson Laboratories. Mice were treated with 150, 100, and 100 mg/kg of CP on days ‐13, ‐11, and ‐9 with respect to the prime vaccination (respectively) via an intraperitoneal injection. Mice were treated with the same dosing regimen again on days 9, 11, and 13. This dosing regimen was chosen due to similarity with the standard regimen when using CP to treat cancer.^35^ A separate cohort of mice were also administered PBS on the same dosing schedule to act as untreated controls.

Male CC mice were obtained in 2021 from the Systems Genetics Core Facility at the University of North Carolina at Chapel Hill.^36^ The 12 strains selected were chosen because they are genetically variable enough to represent the genetic diversity in the human population.^31^

### 2.4 Cell culture

All cells were maintained at 37°C with 5% CO_2_ and ≥95% relative humidity. Lymphocytes were cultured in RPMI 1640 (Corning; Corning, NY) supplemented with 10% fetal bovine serum and 1% penicillin/streptomycin.

### 2.5 Ace‐DEX synthesis

Before synthesis, all glassware was soaked in 1.0 M sodium hydroxide overnight, followed by rinsing with Milli‐Q water to remove any endotoxin contamination. Ace‐DEX was synthesized as previously described.^19^ Briefly, 71 kDa dextran was dissolved in DMSO and then reacted with 2‐ethoxypropene (Matrix Scientific; Montgomery, AL) under anhydrous conditions using pyridinium *p*‐toluenesulfonate as a catalyst. During the reaction, tetrahydrofuran was added to increase the solubility of the reactants. After 2 h, the reaction was quenched with triethylamine (TEA) and the product was precipitated in basic water (0.04% v/v TEA in water). The precipitate was then washed 2x by centrifuging at 21,000*g* for 10 min and then resuspended in basic water. After the washing, the product was dried on a Rotovap to remove any residual solvent and then frozen and lyophilized overnight. The next day, the product was washed another 3x and dried on a Rotovap before freezing and lyophilizing a final time. The cyclic acetal coverage of the final product was analyzed via NMR and determined to be 60% relative cyclic acetal coverage.^19^

### 2.6 MP fabrication

3′3′‐Cyclic GMP‐AMP (cGAMP; Invivogen; San Diego, CA) and J4 COBRA (J4) loaded Ace‐DEX MPs were fabricated by electrospraying.

cGAMP loaded MPs (cGAMP MPs) were fabricated via a monoaxial electrospray apparatus as previously described.^37^ cGAMP and Ace‐DEX were dissolved together in 90% v/v ethanol in water at 20 and 0.2 mg/ml, respectively. This mixture was then loaded into a gas tight glass syringe (Hamilton Company; Reno, NV) with a stainless‐steel cannula attached to the end and placed on a syringe pump (Harvard Apparatus, Holliston, MA). The syringe pump was then positioned vertically over a stainless‐steel collection plate. Finally, the cannula was attached to a negative voltage set to −5 kV, the collection plate was attached to a positive voltage set to +2.5 kV, and the syringe pump was set to 0.2 ml/hr. After the spray was complete, the MPs were collected using a plastic putty knife. cGAMP loading in the MPs was assessed via HPLC.

J4 loaded MPs (J4 MPs) were fabricated via a coaxial electrospray setup similar to as previously described^23^ J4 was dissolved at 8.125 mg/ml in 5% v/v glycerol in water, and Ace‐DEX was dissolved at 25 mg/ml in 70:30 ethyl acetate:butanol. Both solutions were loaded into gas tight glass syringes, placed on syringe pumps, and attached to a custom coaxial spray needle (Rame‐Hart; Succasunna, NJ) with Ace‐DEX in the outer phase and J4 in the inner phase. The needle was then positioned vertically above a stainless‐steel collection plate. The needle was also attached to a negative voltage set to −5 kV while the collection plate was attached to a positive voltage set to +10 kV. To start the spray, the outer phase syringe pump was set to 0.85 ml/h while the inner phase syringe pump was set to 0.02 ml/hr. After the spray was completed, the MPs were collected using a plastic putty knife. J4 loading was measured via the fluorescamine assay as previously described.^38^

The morphology of all the MPs was assessed via SEM (Hitachi s‐4300 Cold Field Emission SEM; Ibaraki, Japan; UNC CHANL). Endotoxin contamination was assessed via the Pierce Chromogenic Endotoxin Quant Kit.

### 2.7 In vivo vaccination studies

For all in vivo vaccination studies, mice were vaccinated on a prime + boost + boost schedule on days 0, 21, and 35. Mice were vaccinated intramuscularly with 25 μl per leg (50 μl total). cGAMP and J4 were both dosed at 1 μg per mouse for all studies. Addavax was chosen as a control given that it is similar to MF59, which is the only adjuvant currently FDA‐approved for non‐pandemic influenza vaccines.^9^ For the Addavax vaccinated groups, Addavax (Invivogen) was mixed 1:1 v/v with J4 and incubated together for at least 20 min in accordance with the manufacturer's protocol.

### 2.8 Determination of antibody titers

Mice were bled on days 14, 28, and 41 via a submandibular bleed. J4‐specific antibody titers in the sera were determined as previously described with a few modifications.^37^ High‐binding 384‐well plates (Greiner Bio‐One; Kremsmünster, Austria) were coated with 25 μl of 100 ng/ml of J4 in PBS overnight. The next day, plates were washed 3x with 50 μl of PBS containing 0.05% v/v Tween‐20 (PBST), and then blocked with 50 μl of 3% v/v instant nonfat dry milk in PBS (Food Lion; Salisbury, NC; blocking buffer) for 1 h. Plates were then washed 3x with PBST and 25 μl of sera diluted 100‐fold (v/v) in blocking buffer followed by 6 5‐fold serial dilutions were added to the wells and incubated for 2 h. Plates were washed 3x with PBST and 25 μl of goat anti‐mouse IgG, IgG1, or IgG2 HRP (Southern Biotech; Birmingham, AL) was added to the wells. After 1 h, the plates were washed 5x with PBST and 25 μl of 3,3′,5,5′‐tetramentylbenzidine was added to the wells and this reaction was quenched with 25 μl of 2 N sulfuric acid. Absorbance was then measured at 450 nm on a SpectraMax M2 microplate reader (Molecular Devices, Sunnyvale, CA), and the absorbance at 570 nm was subtracted from this value to adjust for background. Titer was determined by plotting the serum dilution factor against the background adjusted absorbance and interpolating a cutoff value from this curve as previously described.^39^

### 2.9 Antigen recall experiments

On day 42, mice were humanely euthanized to collect spleens. Spleens were processed into single cells suspensions which were plated at 10^6^ cells/well in a round bottom 96‐well plate and stimulated with 10 μg/ml J4 for 36 h. After 36 h, the supernatant was taken from the cells to measure the antigen specific production of IFN‐γ and IL‐2 via ELISA (Biolegend; San Diego, CA). Cells were also used to assess antigen specific IFN‐γ and IL‐2 via ELISpot (BD Biosciences; San Jose, CA). Spots and cytokine concentration are reported by subtracting the values of the unstimulated controls by the values of the stimulated samples.

### 2.10 Flow cytometry

On day 42, mice were humanely euthanized to collect draining lymph nodes (popliteal + inguinal pooled). Lymphocytes were stained with a fixable viability dye (FVD; eBioscience Fixable Viability Dye eFluor 506) the following fluorescent antibodies from Biolegend (San Diego, CA): CD3 (Alexa Fluor 488, clone: 17A2, concentration: 2 μg/ml), CD4 (APC/Fire 750, clone: RM4‐5, concentration: 1 μg/ml), CD8 (PerCP/Cy5.5, clone: 53–6.7, concentration: 1 μg/ml), CD44 (Brilliant Violet 421, clone: IM7, concentration: 0.5 μg/ml), CD62L (Brilliant Violet 785, clone: MEL‐14, concentration: 0.5 μg/ml), CD19 (APC, clone: 6D5, concentration: 0.5 μg/ml), GL7 (PE, clone: GL7, concentration: 0.125 μg/ml), and CD38 (PE/Cyanine7, clone: 90, concentration: 0.25 μg/ml).

Bone marrow‐derived dendritic cells (BMDCs) were grown as previously described.^40^ After maturation, BMDCs were plated and stimulated with either soluble cGAMP, blank MPs, or cGAMP MPs for 24 h. After 24 h, the supernatant was removed to measure the production of IFN‐β via ELISA (R & D Systems; Minneapolis, MN). Cells were stained with an FVD (eBioscience Fixable Viability Dye eFluor 780) and the following fluorescent antibodies from Biolegend to assess BMDC activation: CD11b (BV711, clone: M1/70, concentration: 0.8 μg/ml), CD11c (BV421, clone: N418, concentration: 1 μg/ml), CD40 (AF647, clone: HM40‐3, concentration: 2 μg/ml), CD80 (PE/Cy7, clone: 16‐10A1, concentration: 2 μg/ml), CD86 (PE, clone: GL‐1, concentration: 2 μg/ml), and MHC‐II (FITC, clone: M5/114.15.2, concentration: 0.8 μg/ml).

After staining and fixing with 1% paraformaldehyde, cells were run on a Thermo Fisher Attune NxT at the UNC Flow Cytometry Core Facility.

### 2.11 Hemagglutination inhibition assay

Hemagglutination inhibition assay (HAI) was run on sera from vaccinated mice as previously described against the following viruses: A/Texas/50/2012(H3N2)(TX/12), A/Switzerland/9715293/2013(H3N2)(SW/13), A/Hong Kong/4801/2014(H3N2)(HK/14), and A/Hong Kong/45/2019(H3N2)(HK/19).^41^

### 2.12 Broad‐sense heritability calculations

Broad‐sense heritability was calculated for different phenotypes across the CC strains by first fitting a linear model: Phenotype ~ CC  + ε. Heritability (H^2^) was then calculated by the following: H^2^ = SSB/(SSB  + SSE) where SSB = sum of squares between and SSE = sum of squares error.^42^

### 2.13 Statistical analysis

Figures were generated using GraphPad Prism 10. Statistics are reported by using an ANOVA followed by Tukey's pairwise comparisons.

## 3 RESULTS AND DISCUSSION

### 3.1 Immunogenicity of J4 and cGAMP MPs in DIO mice

DIO mice were used to investigate the immunogenicity of our broadly active influenza vaccine platform. These mice were not only significantly heavier than healthy control mice, but also had significantly higher serum leptin concentrations and blood glucose concentrations, which are indicative of obesity (Figure S2a–c). For vaccinations, J4 and cGAMP‐loaded Ace‐DEX MPs were fabricated via the highly scalable electrospraying technique, which produced MPs around 1 μm in size with loadings of 11.5 ± 1.4 μg cGAMP/mg MPs and 6.75 ± 0.92 μg J4/mg MPs (Figure S3a,b). MPs also had no detectable levels of endotoxin (<0.1 EU/ml). Non‐DIO and DIO mice were vaccinated on a prime + boost + boost schedule (days 0, 21, and 35) with the following groups: PBS, soluble J4, J4  + Addavax, J4 + cGAMP MPs, or J4 MPs + cGAMP MPs.

**FIGURE 1:**
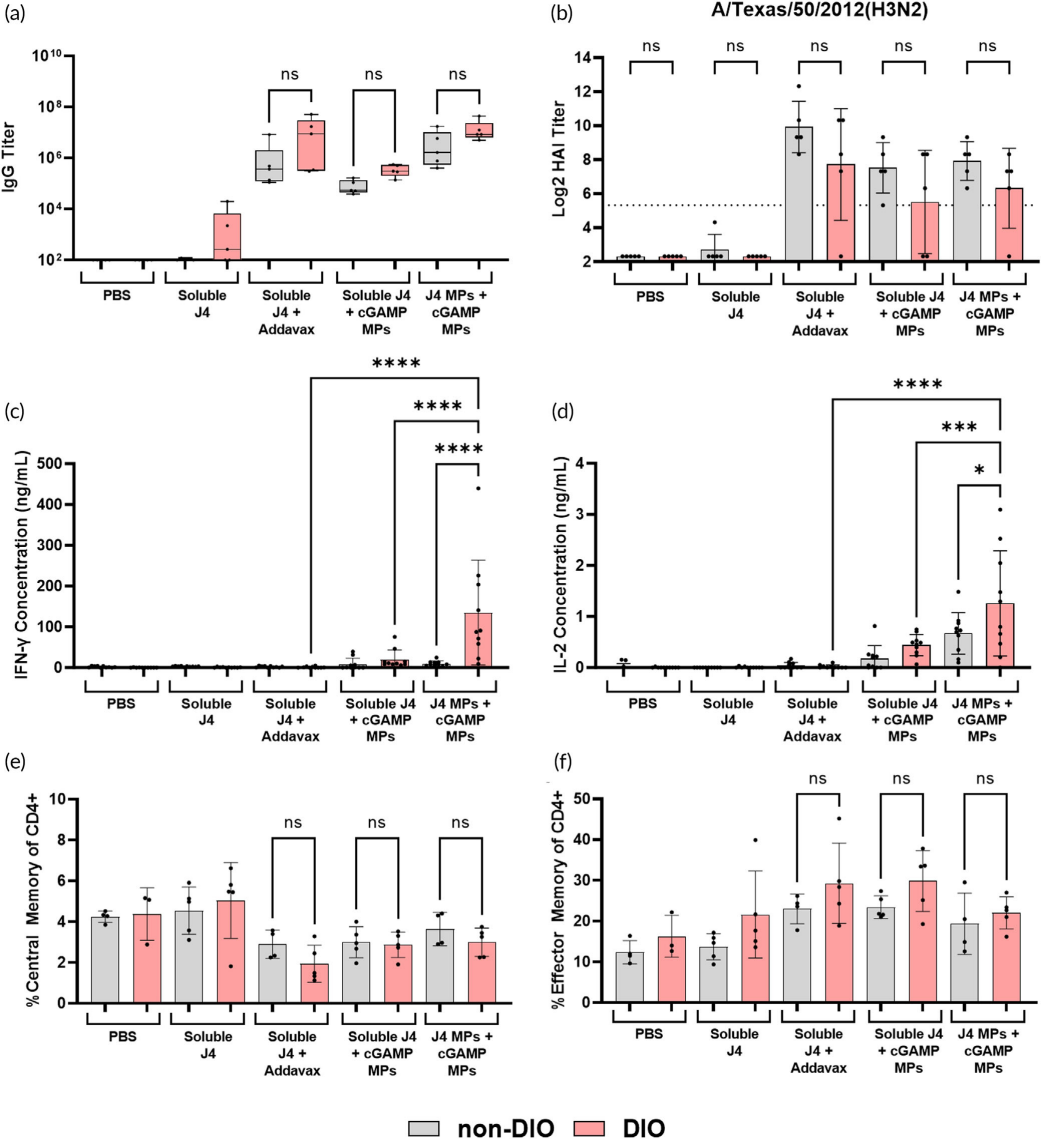
Mice (*n* = 5; non‐DIO or DIO C57BL/6J) were vaccinated on a prime + boost + boost schedule (days 0, 21, and 35) with the indicated groups at doses of 1 μg cyclic GMP‐AMP (cGAMP) and 1 μg J4 per mouse. (a) On day 41, sera were collected and analyzed for J4‐specific IgG titers via ELISA. (b) Day 41 sera was also used to determine HAI activity against A/Texas/50/2012(H3N2). On day 42, mice were humanely euthanized to collect spleens. Splenocytes were stimulated with J4 for 36 h after which the antigen specific production of (c) IFN‐γ and (d) IL‐2 was measured via ELISA. Lymph nodes were also harvested on day 42 to measure the expansion of (e) CD4+ T_CM_ cells (CD3+, CD4+, CD44+, CD62L−) and (f) CD4+ T_EM_ cells (CD3+, CD4+, CD44+, and CD62L+) via flow cytometry. (b) The dotted line represents the seroconversion titer value of 1:40. Data are represented as mean ± SD. ns = *p* > 0.05, * = *p* ≤ 0.05, *** = *p* ≤ 0.001, and **** = *p* ≤ 0.0001. NS means not significant.

We observed no significant differences in J4‐specific IgG titers between the non‐DIO and DIO mice for any of the adjuvanted groups (Figure 1a) (Supplemental Table 1). These trends were consistent with J4‐specific IgG, IgG1, and IgG2c titers for days 14, 28, and 41 (Figure S4a–c). DIO mice vaccinated with cGAMP MPs produced significantly more IgG2c than DIO mice vaccinated with Addavax, indicating cGAMP MPs elicited a more balanced Th1/Th2 immune response induced (Figure S4c). Neutralizing titers, as measured by the HAI, showed similar results to total IgG titers. All adjuvanted groups produced high neutralizing titers across four antigenically distinct influenza viruses, and there were no significant differences between non‐DIO and DIO mice. These results indicate that the COBRA immunogen is just as effective in DIO mice as in non‐DIO mice in producing a broadly neutralizing antibody response (Figure 1b; Figure S5a–c). This broadly active neutralizing antibody response characterized by inhibition of multiple different influenza strains would not be possible with current seasonal vaccinations that only generate substantial neutralizing titers for the specific strains that are included in that season's vaccine.^34^

Our observed humoral response results are surprising given recent studies that have shown a decrease in HAI titer in obese mice vaccinated with an adjuvanted influenza vaccine compared to non‐obese mice.^43^ However, it should be noted that the referenced study used a genetic leptin deficient obese model (Lep^ob^/Lep^ob^) rather than a diet induced model, which could account for this difference in the humoral response. Further, the antibody data herein reveals an advantage of cGAMP MPs as an adjuvant, in that cGAMP MPs produce more IgG2c titers in DIO mice when compared to Addavax while producing similar IgG1 titers as Addavax, and both IgG1 and IgG2 have been shown be important correlates of protection.^44^

We also examined the antigen‐specific cellular immune response in vaccinated DIO and non‐DIO mice at day 42. Splenocytes were collected and stimulated with J4, after which the production of IFN‐γ and IL‐2 were measured. These two cytokines were measured since both IFN‐γ and IL‐2 are produced by Th1 cells, which are not adequately stimulated by the current seasonal influenza vaccines.^45^ DIO mice vaccinated with J4 MPs + cGAMP MPs produced significantly more IFN‐γ and IL‐2 when compared to their non‐DIO counterparts (Figure 1c,d), and this trend was consistent when observing IFN‐γ and IL‐2 ELISpot data (Figure S6a,b). Furthermore, DIO mice vaccinated with J4 MPs + cGAMP MPs produced significantly more IFN‐γ and IL‐2 when compared to DIO mice vaccinated with J4 + cGAMP MPs or J4 + Addavax (Figure 1c,d).

We next directly examined the ability of J4 + cGAMP MPs to elicit an effector T cell response. Following antigen encounter and activation, naïve and preexisting central memory T cells (T_CM_) proliferate and give rise to more highly differentiated effector memory cells (T_EM_).^46^ These effector cells mediate active antigen‐specific cellular immunity, and vaccines that can successfully induce effector T cells are generally more protective.^47^ On day 42 following prime + boost + boost, we examined T cell differentiation state in the lymph nodes of vaccinated DIO and non‐DIO mice. Overall, mice from adjuvanted groups exhibited an increased frequency of T_EM_ cells and a decreased frequency of T_CM_ cells (Figure 1e,f). There were no significant differences in these frequencies when comparing DIO and non‐DIO mice (Figure 1e,f), indicating that obesity does not inhibit cGAMP‐adjuvanted T cell priming and differentiation (1E‐F). The expansion of germinal center (GC) B cells was also examined, and much like the memory T cell phenotypes there was no significant difference between DIO and non‐DIO mice (Figure S7). Cumulatively, these results show that DIO mice have an enhanced antigen recall response to J4 and cGAMP loaded MPs when compared to non‐DIO mice (Figure 1c,d). These data also indicate that the encapsulation of J4 in Ace‐DEX MPs enhances the cellular immune response in DIO mice when compared to unencapsulated J4 (Figure 1c,d).

**FIGURE 2:**
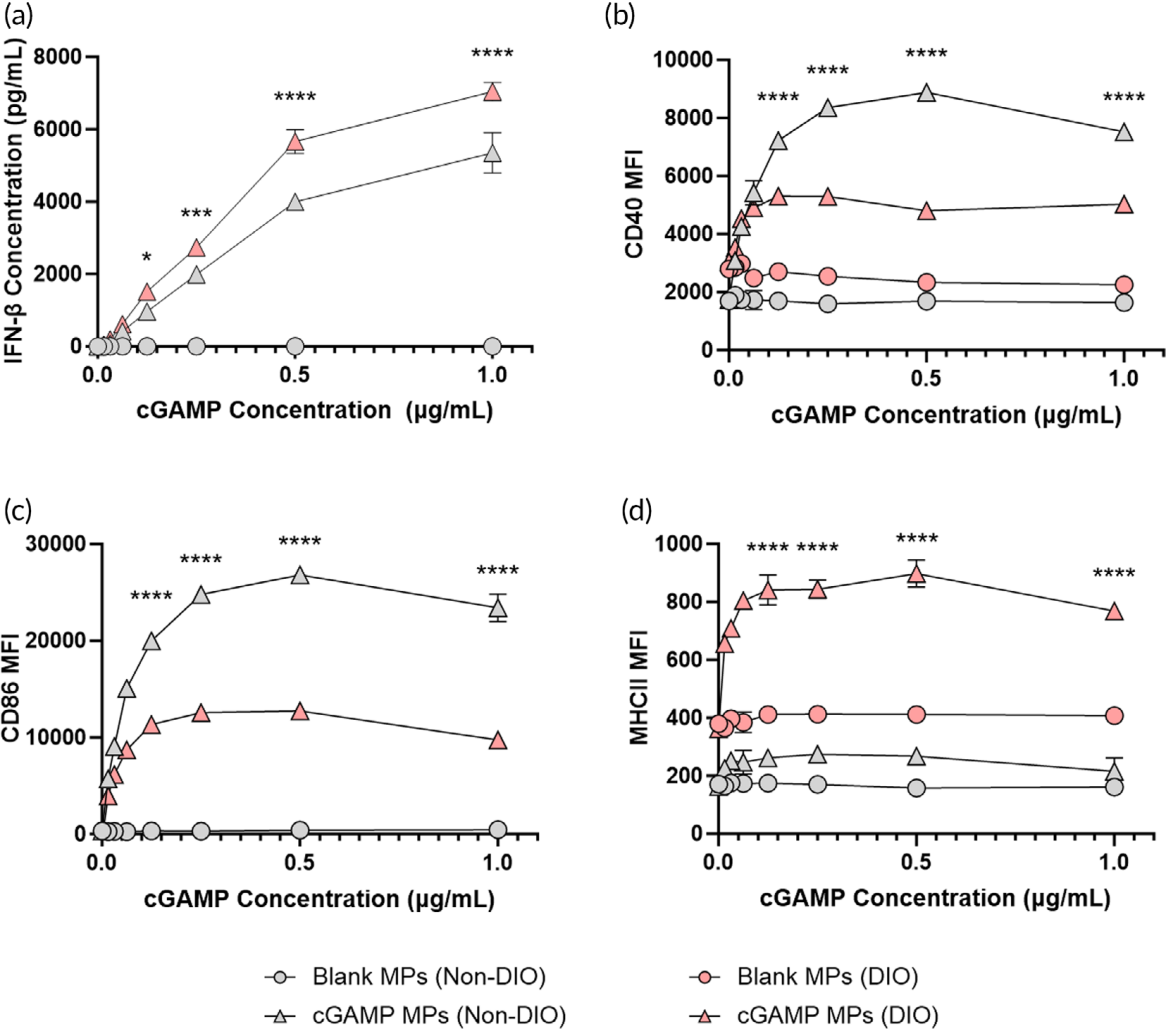
Bone marrow was harvested from non‐diet induced obese (DIO) or DIO mice and differentiated into bone marrow‐derived dendritic cells (BMDCs). BMDCs were stimulated for 24 h with blank microparticles or cyclic GMP‐AMP (cGAMP) MPs. (a) After 24 h, the production of IFN‐β was measured via ELISA. Surface expression of (b) CD40, (c) CD86, and (d) MHCII was also measured via flow cytometry. (b–d) Cells are gated on CD11b+ and CD11c+. Statistics represent comparisons between cGAMP MPs (non‐DIO) and cGAMP MPs (DIO). Data are represented as mean ± SD. * = *p* ≤ 0.05, *** = *p* ≤ 0.001, and **** = *p* ≤ 0.0001.

Next, to determine whether the increased cellular response in DIO mice was due to differences in innate immune function, bone marrow was taken from both DIO and non‐DIO mice and differentiated into BMDCs. The BMDCs were stimulated with cGAMP MPs or blank MPs for 24 h. Compared to non‐DIO BMDCs, cGAMP MPs produced significantly more IFN‐β and caused significantly higher expression of MHC II in DIO BMDCs (Figure 2a–d). Conversely, cGAMP MPs caused lower expression of CD40 and CD86 in DIO BMDCs when compared to non‐DIO BMDCs (Figure 2b,c). These results suggest that the improved cellular response of DIO mice could result from increased IFN‐β and MHC‐II expression following STING activation. While lower CD40 and CD86 expression did not lead to decreased vaccine efficacy, this may be because these costimulatory markers are expressed above a certain threshold for activation and did not affect the immunogenicity as much as IFN‐β and MHC‐II. It is important to recognize limitations of this experiment in that these in vitro results may not fully capture an in vivo system. Due to this, future work can include the investigation of the in vivo activation of APCs in DIO versus non‐DIO mice.

Taken together, these results demonstrate that cGAMP‐ and J4‐loaded MPs produce a potent and broadly active humoral and cellular immune response in DIO mice. This response is enhanced compared to non‐DIO mice vaccinated with cGAMP and J4 loaded MPs and DIO mice vaccinated with soluble J4 adjuvanted with cGAMP MPs or soluble J4 with Addavax. This suggests that these MPs could be a promising platform for the protection of obese populations from influenza when compared to a clinically approved adjuvant; however, future work needs to be conducted to assess protection after challenge in this mouse model.

### 3.2 Immunogenicity of J4 and cGAMP MPs in aged mice

**FIGURE 3:**
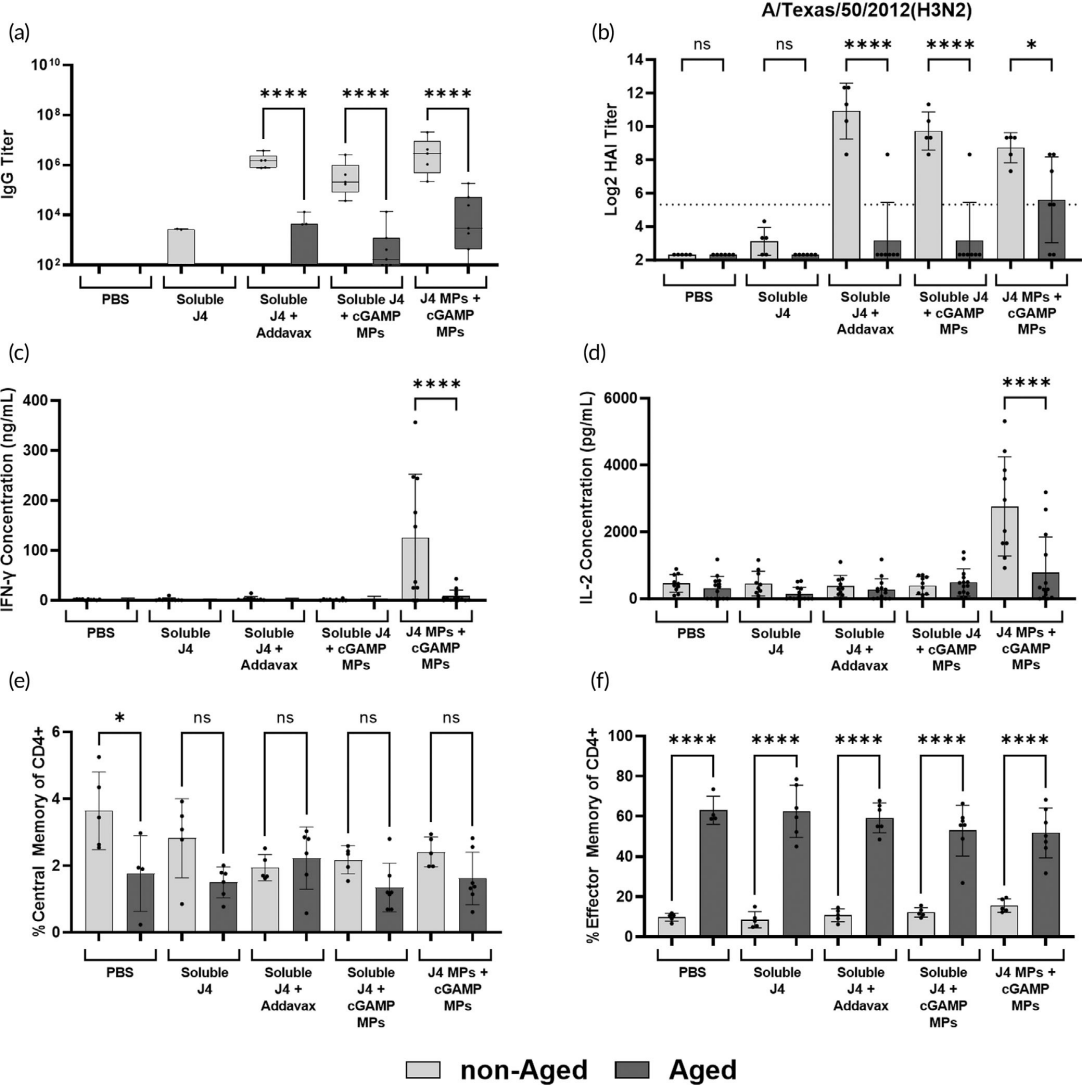
Mice (*n* = 5 non‐aged or *n* = 7 aged C57BL/6J) were vaccinated on a prime + boost + boost schedule (days 0, 21, and 35) with the indicated groups at doses of 1 μg cyclic GMP‐AMP (cGAMP) and 1 μg J4 per mouse. (a) On day 41, sera were collected and analyzed for J4‐specific IgG titers via ELISA. (b) Day 41 sera was also used to determine HAI activity against A/Texas/50/2012(H3N2). On day 42, mice were humanely euthanized to collect spleens. Splenocytes were stimulated with J4 for 36 h after which the antigen specific production of (c) IFN‐γ and (d) IL‐2 was measured via ELISA. Lymph nodes were also harvested on day 42 to measure the expansion of (e) CD4+ T_CM_ cells (CD3+, CD4+, CD44+, CD62L−) and (f) CD4+ T_EM_ cells (CD3+, CD4+, CD44+, and CD62L+) via flow cytometry. (b) The dotted line represents the seroconversion titer value of 1:40. Data are represented as mean ± SD. ns = *p* > 0.05, * = *p* ≤ 0.05, *** = *p* ≤ 0.001, and **** = *p* ≤ 0.0001. NS means not significant.

Given the enhancement of the cellular immune response produced by J4 and cGAMP MPs in obese mice, we were curious about the immunogenicity of our platform in aged mice, another immunocompromised population. Additionally, current clinically approved seasonal vaccines have been shown to be less immunogenic in aged populations.^4^ To this end, 18‐month‐old mice along with 6‐ to 8‐week‐old controls were vaccinated with the following groups: PBS, soluble J4, J4 + Addavax, J4 + cGAMP MPs, or J4 MPs + cGAMP MPs. First, the humoral response of these mice was investigated. Day 41 J4‐specific IgG titers were lower for the aged mice compared to the non‐aged mice for all adjuvanted groups (Figure 3a) (Supplemental Table 2). This was also true for day 14, 28, and 41 J4‐specific IgG, IgG1, and IgG2c titers (Figure S8a–c). When we examined the neutralizing antibody response via HAI, the results were similar to that of the total anti‐J4 IgG titer. Aged mice had significantly lower HAI titers across all four viruses assayed for the three adjuvanted groups (Figure 3b; Figure S9a–c). J4 MPs + cGAMP MPs did cause seroprotection (HAI titer ≥1:40) in a few of the aged mice against TX/12 and HK/14, whereas mice vaccinated with soluble J4 with Addavax or cGAMP MPs did not have as many mice seroconvert (Figure 3b; Figure S9a–c; indicated with dotted line). These results indicate that both Addavax and cGAMP MPs are unable to produce a significant humoral response against J4 in aged mice.

To determine the cellular immune response in these mice, a similar experiment was conducted as above where splenocytes from vaccinated mice were stimulated with J4 for 36 h to measure the antigen specific production of IFN‐γ and Il‐2. Analysis by both ELISA and ELISpot showed that non‐aged mice vaccinated with J4 MPs + cGAMP MPs produced significantly more IFN‐γ and IL‐2 when compared to aged mice also vaccinated with J4 MPs + cGAMP MPs (Figure 3c,d; Figure S10a,b). To investigate the potential mechanism behind the decreased cellular immune response, lymph nodes were taken to measure the expansion of CD4+ T_EM_ and T_CM_ cells. Just as with the obese and non‐obese mice, the non‐aged mice showed a decreased frequency of CD4+ T_CM_ cells with a corresponding increased frequency of CD4+ T_EM_ cells (Figure 3e,f). Comparing non‐aged to aged mice, there was no difference in the frequency of CD4+ T_CM_ cells for any of the adjuvanted groups (Figure 3e). Conversely, there was a significant increase in the frequency of CD4+ T_EM_ cells in the aged mice compared to the non‐aged mice across all groups (Figure 3f). Further, there were significant decreases in the total numbers of CD4+ and CD8+ T cells in the lymph nodes of aged mice compared to non‐aged mice across all groups (Figure S11b,c). The expansion of GC B cells was similar between non‐aged and aged mice across all groups (Figure S11a). Overall, these results corroborate the results of the antigen recall experiments. The lack of a significant antigen recall response in the aged mice was likely due to a significantly smaller naïve T cell pool as seen by a higher frequency of CD4+ T_EM_ cells in the aged mice (Figure 3f). This phenomenon is likely due to thymic involution which is a well reported process in aging that decreases the naïve T cell pool.^48^ These data are also similar to the humoral response results in that J4 MPs + cGAMP MPs are unable to save the cellular immune response in aged mice when compared to their non‐aged counterparts.

**FIGURE 4:**
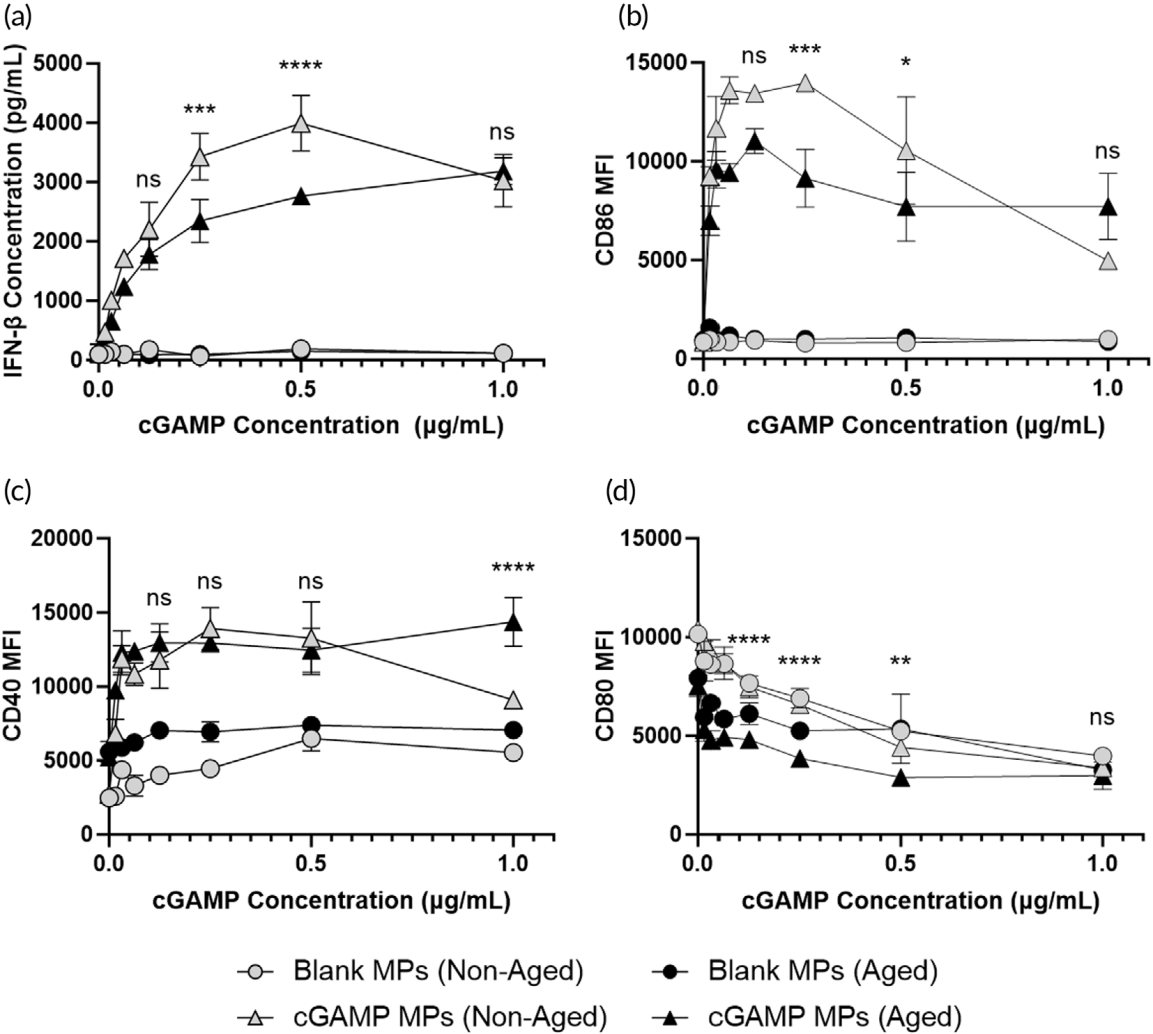
Bone marrow was harvested from non‐aged or aged mice and differentiated into bone marrow‐derived dendritic cells (BMDCs). BMDCs were stimulated for 24 h with blank microparticles (MPs) or cyclic GMP‐AMP (cGAMP) MPs. (a) After 24 h, the production of IFN‐β was measured via ELISA. Surface expression of (b) CD40, (c) CD86, and (d) MHCII was also measured via flow cytometry. (b–d) Cells are gated on CD11b+ and CD11c+. Statistics represent comparisons between cGAMP MPs (non‐DIO) and cGAMP MPs (DIO). Data are represented as mean ± SD. * = *p* ≤ 0.05, *** = *p* ≤ 0.001, and **** = *p* ≤ 0.0001. NS means not significant.

To determine whether the significant decreases in the cellular and humoral immune responses to J4 MPs + cGAMP MPs in aged mice were due to a deficiency in innate immunity, non‐aged and aged BMDCs were stimulated with blank MPs and cGAMP MPs. cGAMP MPs produced a similar amount of IFN‐β in non‐aged and aged BMDCs except at 0.5 and 0.25 μg cGAMP/ml, where the non‐aged BMDCs produced significantly more IFN‐β than the aged BMDCs (Figure 4a). When we examined the expression of costimulatory markers, the non‐aged and aged BMDCs behaved similarly after stimulation with cGAMP MPs. CD86 and CD40 expressions were not significantly different for non‐aged and aged BMDCs after cGAMP stimulation except at 0.25 and 0.5 μg cGAMP/ml for CD86 and at 1 μg cGAMP/ml for CD40 (Figure 4b,c). MHC‐II expression was significantly different between non‐aged and aged BMDCs when stimulated with cGAMP MPs except at 1 μg cGAMP/ml, which was not significantly different (Figure 4d). These data indicate that the innate immune function of the aged mice may have been slightly dampened compared to the non‐aged mice; however, the more likely reason for the decreased antigen recall activity is the smaller naïve T cell pool (Figure 3f). It should be noted that this in vitro experiment may not fully capture the activation of APCs in vivo, so future work can include the investigation of the in vivo activation of APCs in aged versus non‐aged mice.

These results indicate that both Addavax and cGAMP MPs are unable to promote a strong humoral or cell mediated immune response in aged mice. Though there have been many reports of STING agonists used as vaccine adjuvants, there has been little investigation of the efficacy of these adjuvants in aged populations. A recent study from Vassilieva et al. showed that a STING agonist combined with Quil‐A was able to protect aged mice against a lethal influenza challenge with an enhanced cellular immune response.^49^, ^50^ Conversely, in this study, cGAMP MPs alone were unable to promote a significant cellular or humoral immune response against the J4 antigen in aged mice. It should be noted that Vassilieva et al. investigated their platform in BALB/c mice (a Th2‐skewing strain), whereas the present study was conducted in C57BL/6 (a Th1‐skewing strain), suggesting that there could be a strain dependency on the efficacy of STING agonists in aged mice. Furthermore, the decreased IFN‐β production reported in the present manuscript corroborates findings of STING dysfunction in aged mice after bacterial infection and other reports of general STING dysregulation in aged populations.^51^, ^52^ Based on these results, future work could include combining cGAMP with other adjuvants to promote a stronger immune response. The dosing of both J4 and cGAMP could also be explored since there has been success of the high dose Fluzone in aged populations.

### 3.3 Immunogenicity of J4 and cGAMP MPs in mice treated with chemotherapy

**FIGURE 5:**
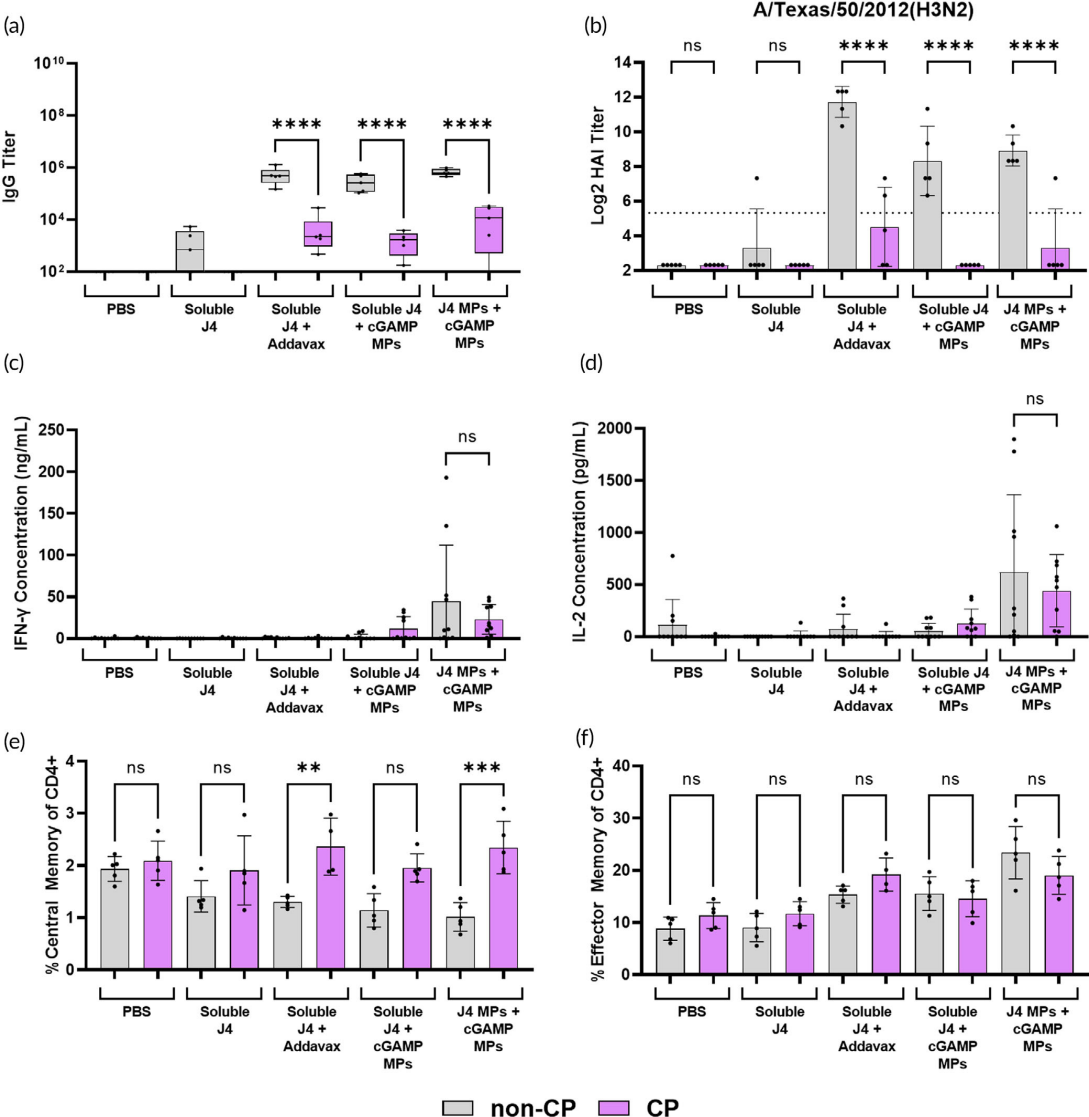
Mice (*n* = 5; non‐cyclophosphamide treated [non‐CP] or cyclophosphamide treated [CP] C57BL/6J) were vaccinated on a prime + boost + boost schedule (days 0, 21, and 35) with the indicated groups at doses of 1 μg cGAMP and 1 μg J4 per mouse. (a) On day 41, sera were collected and analyzed for J4‐specific IgG titers via ELISA. (b) Day 41 sera was also used to determine HAI activity against A/Texas/50/2012(H3N2). On day 42, mice were humanely euthanized to collect spleens. Splenocytes were stimulated with J4 for 36 h after which the antigen specific production of (c) IFN‐γ and (d) IL‐2 was measured via ELISA. Lymph nodes were also harvested on day 42 to measure the expansion of (e) CD4+ T_CM_ cells (CD3+, CD4+, CD44+, CD62L−) and (f) CD4+ T_EM_ cells (CD3+, CD4+, CD44+, and CD62L+) via flow cytometry. (b) The dotted line represents the seroconversion titer value of 1:40. Data are represented as mean ± SD. ns = *p* > 0.05, ** = *p* ≤ 0.01, *** = *p* ≤ 0.001, and **** = *p* ≤ 0.0001. NS means not significant.

Similar to obese and aged populations, there have also been reports that seasonal influenza vaccines are less effective for individuals undergoing chemotherapy.^53^ To test whether our vaccine platform could improve efficacy in this population, mice were treated with a regimen of the chemotherapy CP with a schedule and dosing akin to what is used in the clinic to treat a variety of cancers such as lymphoma, leukemia, breast cancer, and ovarian cancer.^35^ CP has also been shown to cause immunosuppression by killing immune cells.^54^ Mice treated with CP and control mice (non‐CP) were vaccinated with the following groups: PBS, soluble J4, J4 + Addavax, J4 + cGAMP MPs, or J4 MPs + cGAMP MPs. First, the humoral response in this model was measured. J4‐specific IgG titers were significantly reduced across all of the adjuvanted groups for CP mice compared to non‐CP mice at day 41 (Figure 5a) (Supplemental Table 3). This was also true for J4‐specific IgG, IgG1, and IgG2c on days 14, 28, and 41 (Figure S12a–c). Results for HAI were similar to that of the IgG titers. For all four viruses examined, CP mice had significantly lower HAI titers when compared to non‐CP mice across the three adjuvanted groups (Figure 5b; Figure S13a–c). These data demonstrate that CP reduces the efficacy of both Addavax and cGAMP MPs in generating a humoral response against J4.

Since cellular immunity is another mode of protection against influenza, we investigated whether the cellular response was maintained in mice treated with CP. As above, splenocytes were stimulated with J4, after which the production of IFN‐γ and IL‐2 was measured via ELISA and ELISpot. There was no significant difference in IFN‐γ and IL‐2 production between non‐CP and CP mice vaccinated with J4 MPs + cGAMP MPs (Figure 5CD; Figure S14a,b). Further, ELISpot showed that both the non‐CP and CP mice vaccinated with J4 MPs + cGAMP MPs produced significantly more IFN‐γ secreting cells when compared to their counterparts vaccinated with J4 + Addavax (Figure S14a). To further investigate this antigen recall data, the expansion of memory T cells in the lymph nodes was examined. Generally, CP mice had a higher frequency of T_CM_ cells for the adjuvanted groups (Figure 5e). This could indicate that these cells are not efficiently differentiating into T_EM_ cells after antigenic restimulation. However, there were no significant differences in T_EM_ cell frequencies between non‐CP and CP mice for the adjuvanted groups (Figure 5f). There were also no significant differences between non‐CP and CP mice in the expansion of GC B cells in the adjuvanted groups (Figure S15a). Finally, CP mice showed lower counts of both CD4+ and CD8+ T cells when compared to non‐CP mice (Figure S15b,c).

These data indicate the utility of J4 and cGAMP loaded MPs over Addavax in healthy and CP‐treated mice. While neither soluble J4 + Addavax nor J4 MPs + cGAMP MPs could not mount a substantial humoral response in CP mice (Figure 5a,b), J4 MPs + cGAMP MPs gave the same antigen recall response in non‐CP and CP mice (Figure 5c,d). This provides a promising platform for the vaccination of individuals on chemotherapy who normally do not respond well to vaccines.^7^ It should be noted that the CP mice did have lower numbers of CD4+ and CD8+ T cells, so whether the antigen recall response demonstrated herein is enough to protect against infection is a question for future research (Figure S15b,c). Future work in this model could also investigate whether vaccinating before the start of the chemotherapy regimen influences the immunogenicity of the vaccine.

### 3.4 Immunogenicity of J4 and cGAMP MPs in the CC mice

While mouse models of obesity, aging, and chemotherapy can capture specific cases of immunocompromising conditions, they do not sufficiently capture the diversity of the human population that will receive a broadly active influenza vaccine. To evaluate cGAMP MPs in a genetically diverse population, CC mice were used. Twelve different CC strains were vaccinated on a prime + boost + boost schedule with either soluble J4 + Addavax or Soluble J4 + cGAMP MPs.

**FIGURE 6:**
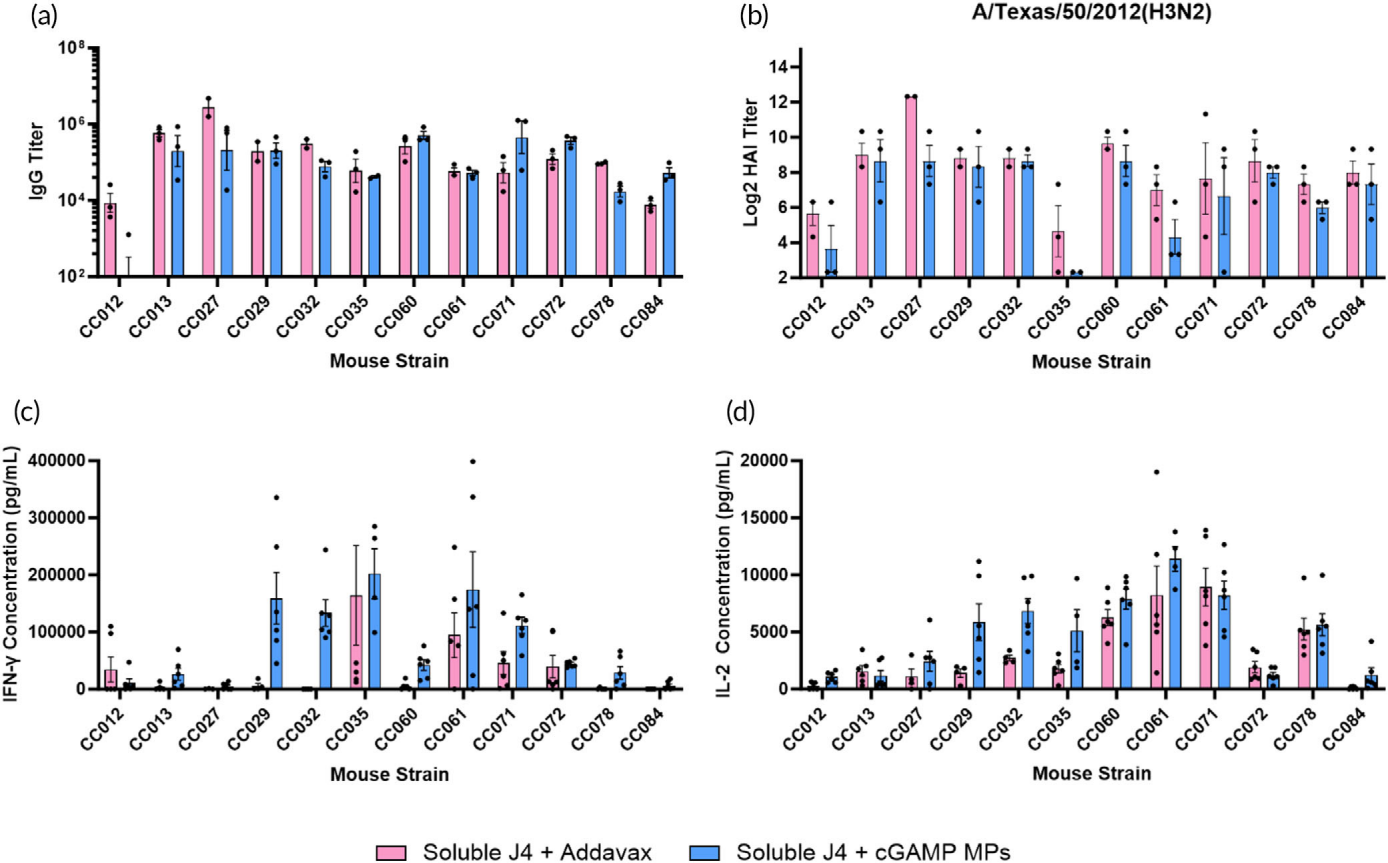
Mice (*n* = 3; 12 different collaborative cross strains) were vaccinated on a prime + boost + boost schedule (days 0, 21, and 35) with the indicated groups at doses of 1 μg cGAMP and 1 μg J4 per mouse. (a) On day 41, sera were collected and analyzed for J4‐specific IgG titers via ELISA. (b) Day 41 sera was also used to determine HAI activity against A/Texas/50/2012(H3N2). On day 42, mice were humanely euthanized to collect spleens. Splenocytes were stimulated with J4 for 36 h after which the antigen specific production of (c) IFN‐γ and (d) IL‐2 was measured via ELISA. Data are represented as mean ± SD.

First, the humoral response of these mice was examined. Whether vaccinated with Addavax or cGAMP MPs, all CC strains produced a robust anti‐J4 IgG response except for CC012 which only produced detectable anti‐J4 IgG titers when vaccinated with Addavax (Figure 6a) (Supplemental Table 4). Outside of total IgG, it is important to consider IgG subtype production since both IgG1 and IgG2 subtypes have been shown to play separate roles in protection against influenza.^44^ Much like total anti‐J4 IgG titers, mice vaccinated with Addavax or cGAMP MPs were both able to produce a robust IgG1 response (Figure S16a). For IgG2ac, CC mice vaccinated with cGAMP MPs produced a more robust response. For example, CC084, CC027, and CC072 all had improved IgG2ac titers on average when compared to the same mice vaccinated with Addavax indicating a more Th1 skewed immune response (Figure S16b). For neutralizing titers, both Addavax and cGAMP MPs were mostly able to produce high titers across the three examined viral strains; however, there were some low responding CC strains. For example, CC035 produced higher TX/12 and HK/14 neutralizing titers on average when vaccinated with Addavax compared to cGAMP MPs. This humoral response data show that both Addavax and cGAMP MPs produce a broadly neutralizing antibody response in a genetically diverse population (Figure 6b; Figure S16c,d). Further, cGAMP has the benefit over Addavax of producing a more robust IgG2ac response across the CC strains tested (Figure S16b).

Finally, the antigen recall response of these mice against J4 was evaluated via the same assay as above. Overall, CC mice vaccinated with cGAMP MPs produced a much more robust antigen specific IFN‐γ response when compared to Addavax vaccinated mice. CC029, CC032, CC061, and CC078 all produced more IFN‐γ on average when vaccinated with cGAMP MPs compared to the same mice vaccinated with Addavax (Figure 6c; Figure S17a). IL‐2 production was more similar between cGAMP MPs and Addavax vaccinated mice except for CC029 and CC032 which produced more IL‐2 on average when vaccinated with cGAMP MPs (Figure 6d; Figure S17b).

To determine the overall variability observed in each vaccine response (e.g., IgG titer, IFN‐γ production after antigen recall) attributable to inherited genetic factors, heritability was calculated (Supplemental Table 5). For anti‐J4 IgG titer, the heritability was lower for cGAMP MP vaccinated mice compared to Addavax vaccinated mice. However, for anti‐J4 IgG1 and IgG2ac the heritability was higher for cGAMP MP vaccinated mice. Heritability calculations for the cellular response revealed that cGAMP MP vaccinated mice had higher heritability for IFN‐γ production after antigen recall, but lower heritability for IL‐2 production after antigen recall. These heritability calculations support future studies investigating crossbreeding of these CC strains to determine which genetic factors are causing high heritability of the different vaccine responses.

These results indicate that cGAMP MPs can produce a more robust cellular response in a genetically diverse population of mice when compared to Addavax. There is a significant amount of literature describing the importance of cell mediated immunity in protection against influenza, so this finding further highlights the translational potential of cGAMP MPs as an adjuvant for a broadly active influenza vaccine.^55^ It should be noted that there were some CC strains that did not have a strong cellular response to cGAMP MPs or Addavax, such as CC027 (Figure 6c). It is likely that this low response is caused by differential expression of a genetic locus that is highly heritable, as shown with the heritability of the IFN‐γ response (Supplemental Table 1). The cellular response in these strains may also be improved by the encapsulation of J4 in Ace‐DEX MPs as seen with the other immunocompromised populations above, so future work could investigate vaccinating CC strains with J4 MPs and cGAMP MPs. Challenge in the CC model after vaccination would also be an interesting avenue for future investigation, as previous work has demonstrated a diverse range in severity of disease pathology across CC strains after influenza challenge.^56^ Furthermore, to date there has been no investigation of the differential expression or polymorphisms of STING across CC strains. It is well understood that STING polymorphisms can greatly affect STING agonism in humans, so future research in this area is warranted to determine whether cGAMP MPs also show differential activity across human STING polymorphisms.^57^

## 4 CONCLUSION

The present work investigated the immunogenicity of a COBRA HA (J4)‐based broadly active influenza vaccine adjuvanted with cGAMP‐loaded Ace‐DEX MPs in several clinically relevant mouse models, including obese, aged, chemotherapy‐induced immunosuppression, and genetically diverse population populations. MPs and cGAMP MPs produced a humoral response in obese mice that closely mimicked that observed in non‐obese mice while providing an enhanced cellular response in obese mice when compared to Addavax. The cGAMP MP adjuvant was not as effective in aged mice, where both the humoral and cellular immune responses were dampened compared to young controls. Further analysis suggested that these results could be due to thymic involution and age‐related STING dysfunction. In mice on a chemotherapy regimen, cGAMP MPs produced a strong antigen recall response that was not significantly different from untreated control mice. Finally, while Addavax and cGAMP MPs both produced a robust humoral response in CC mice, cGAMP MPs produced a more robust cellular response in the different CC strains examined. The data presented herein could be limited by the use of single sex mice for each model, since recent literature has shown sex can have an impact on immune responses.^58^, ^59^ Future work can include investigating whether the sex of these models changes the response to our vaccine platform.

In conclusion, these results indicate that while cGAMP MPs are effective in some immunocompromised models, such as obesity and chemotherapy‐induced immunosuppression, they are not as effective in others, such as aging. Further, cGAMP MPs were not effective in some of the CC strains examined. This supports work in the field of precision vaccinology, where adjuvants are specifically tailored to vulnerable populations with distinct immunological deficits. In line with this, future work could include combining cGAMP MPs with other adjuvants that could be more immunogenic in populations where STING signaling may be dysfunctional and evaluating these systems in an influenza challenge model. Future studies should aim to in protecting those who are most at risk for influenza virus infection and inform better vaccine design for those populations.

## 5 SUPPLEMENTAL DATA

Figure S1 is a schematic of the breeding strategy for the CC. Figure S2 contains SEM micrographs of the MPs used throughout the manuscript. Figure S3 contains the weight, serum leptin concentrations, and blood glucose concentrations of mice on the control or high‐fat diet. Supplemental Table 1 contains *p*‐values for comparisons not shown in Figure 1. Figure S4 contains IgG, IgG1, and IgG2c titers for days 14, 28, and 41 for the obese study. Figure S5 contains HAI titers for the obese study against three additional viruses. Figure S6 contains IFN‐γ and IL‐2 ELISpot data for the obese study antigen recall. Figure S7 contains the expansion of GC B cells for the obese study. Supplemental Table 7 contains *p*‐values for comparisons not shown in Figure 3. Figure S8 contains IgG, IgG1, and IgG2c titers for days 14, 28, and 41 for the aged study. Figure S9 contains HAI titers for the aged study against three additional viruses. Figure S10 contains IFN‐γ and IL‐2 ELISpot data for the aged study antigen recall. Figure S11 contains GC B cell frequency, CD4+ T cell counts, and CD8+ T cell counts for the aged study. Supplemental Table 3 contains *p*‐values for comparisons not shown in Figure 5. Figure S12 contains IgG, IgG1, and IgG2c titers for days 14, 28, and 41 for the cyclophosphamide study. Figure S13 contains HAI titers for the cyclophosphamide study against three additional viruses. Figure S14 contains IFN‐γ and IL‐2 ELISpot data for the cyclophosphamide study antigen recall. Figure S15 contains GC B cell frequency, CD4+ T cell counts, and CD8+ T cell counts for the cyclophosphamide study. Supplemental Table 4 contains *p*‐values for comparisons not shown in Figure 6. Figure S16 contains IgG1 and IgG2ac titers as well as HAI against two additional viruses for the CC study. Figure S17 contains IFN‐γ and IL‐2 ELISpot data for the CC study antigen recall. Supplemental Table 5 contains heritability values for the different vaccination endpoints measured in the CC study.

## AUTHOR CONTRIBUTIONS

Conceptualization (Kristy M. Ainslie, Eric M. Bachelder, Dylan A. Hendy). Data curation (Dylan A. Hendy). Formal analysis (Dylan A. Hendy, Erik S. Pena, Luis Ontiveros‐Padilla). Funding acquisition (Kristy M. Ainslie, Ted M. Ross). Investigation (Dylan A. Hendy, Erik S. Pena, Luis Ontiveros‐Padilla, Timothy A. Dixon, Denzel D. Middleton, Grace L. Williamson, Nicole Rose Lukesh, Sean R. Simpson, Rebeca T. Stiepel, Md Jahirul Islam, Michael A. Carlock). Methodology (Dylan A. Hendy, Erik S. Pena, Luis Ontiveros‐Padilla, Michael A. Carlock). Project administration (Kristy M. Ainslie, Ted M. Ross, Dylan A. Hendy). Resources (Michael A. Carlock). Supervision (Kristy M. Ainslie, Eric M. Bachelder, Ted M. Ross). Validation (Dylan A. Hendy). Visualization (Dylan A. Hendy, Erik S. Pena, Luis Ontiveros‐Padilla). Writing (Dylan A. Hendy). Reviewing and editing (Erik S. Pena, Luis Ontiveros‐Padilla, Ted M. Ross, Eric M. Bachelder, Kristy M. Ainslie).

## FUNDING INFORMATION

This work was supported, in part, by National Institutes of Health (NIH) NIAID Collaborative Influenza Vaccine Innovation Centers (CIVICs) Contract #75N93019C00052 (PI: Ross), NIH R01AI147497 (PI: Ainslie), and UNC Systems Genetics Core Facility Collaborative‐Cross Program Special Pilot 21‐0112 (PI: Ainslie). The UNC Flow Cytometry Core Facility (RRID:SCR_019170) is supported in part by P30 CA016086 Cancer Center Core Support Grant to the UNC Lineberger Comprehensive Cancer Center.

## CONFLICT OF INTEREST STATEMENT

The authors declare no conflicts to declare.

### PEER REVIEW

The peer review history for this article is available at https://www.webofscience.com/api/gateway/wos/peer-review/10.1002/btm2.10634.

## Supporting information


**DATA S1:** Supplementary Information.

## Data Availability

The data that support the findings of this study are available from the corresponding author upon reasonable request.
