# Supplementary material for: Immunogenicity of an adjuvanted broadly active influenza vaccine in immunocompromised and diverse populations
Source: Bioeng Transl Med. 2023 Dec 8;9(2):e10634. doi: 10.1002/btm2.10634 (PMC10905549; doi:10.1002/btm2.10634)
Supplement: Supplementary file 1 — DATA S1: Supplementary Information. [file BTM2-9-e10634-s001.docx]

**Supplementary Data: Immunogenicity of a cGAMP Microparticle Adjuvanted Broadly Active Influenza Vaccine in Immunocompromised and Diverse Populations**

Dylan A. Hendy^a^, Erik S. Pena^b^, Luis Ontiveros-Padilla^a^, Timothy A. Dixon^a^, Denzel D. Middleton^a^, Grace L. Williamson^a^, Nicole Rose Lukesh^a^, Sean R. Simpson^a^, Rebeca T. Stiepel^a^, Md Jahirul Islam^a^, Michael A. Carlock^c^, Ted M. Ross^cde^, Eric M. Bachelder^a^, and Kristy M. Ainslie^abf^*

^a^ Division of Pharmacoengineering and Molecular Pharmaceutics, Eshelman School of Pharmacy, University of North Carolina at Chapel Hill, USA.

^b^ Joint Department of Biomedical Engineering, University of North Carolina at Chapel Hill and North Carolina State University, USA.

^c^ Florida Research and Innovation Center, Port Saint, Cleveland Clinic Florida, Port St. Lucie, FL, USA

^d^ Center for Vaccines and Immunology, University of Georgia, Athens, GA, USA.

^e^ Department of Infectious Diseases, University of Georgia, Athens, GA, USA.

^f^ Department of Microbiology and Immunology, UNC School of Medicine, University of North Carolina, Chapel Hill, NC, USA.

*Corresponding Author

Kristy M. Ainslie, PhD

Fred Eshelman Distinguished Professor

Chair, Division of Pharmacoengineering & Molecular Pharmaceutics

UNC Eshelman School of Pharmacy

4012 Marsico Hall, 125 Mason Farm Road

Chapel Hill, NC 27599, United States

[ainsliek@email.unc.edu](mailto:ainsliek@email.unc.edu)


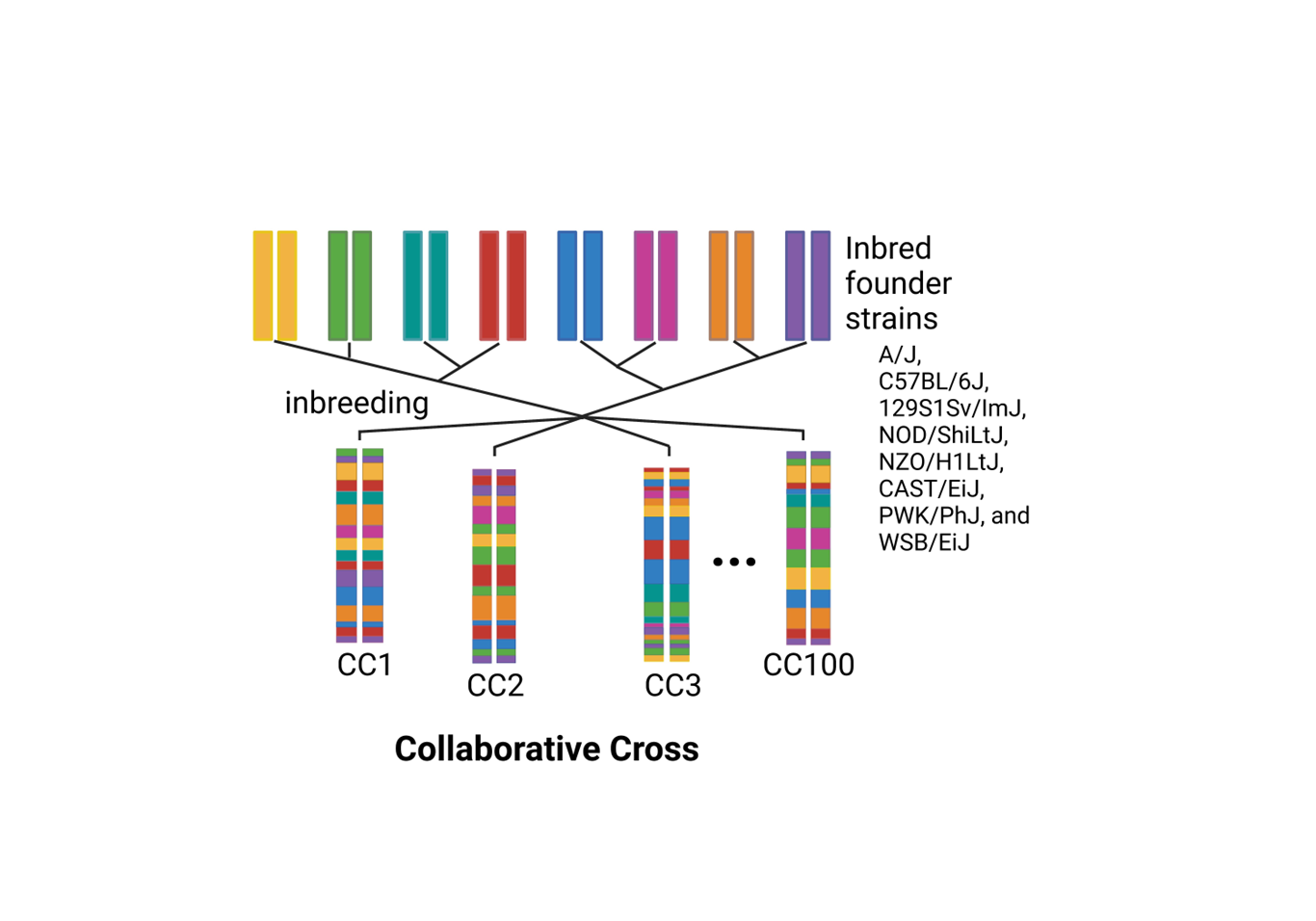


**Figure S1.** General schematic of the method by which the collaborative cross (CC) mice were bred.

**
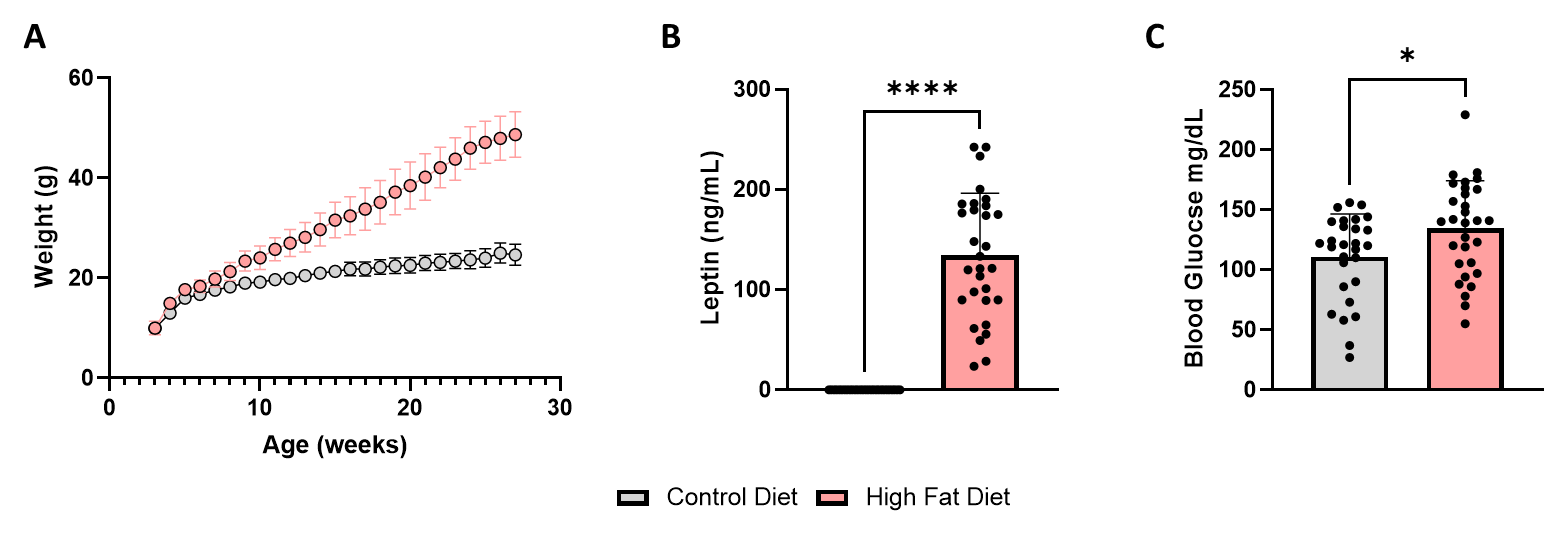
**

**Figure S2.** Starting at 3 weeks of age, C57BL/6J (n = 30) were fed either a control diet or a high fat diet until the completion of the vaccination study at 28 weeks. (A) Mouse weight was monitored weekly for the entire study. (B) On week 19 mice were bled, and sera leptin levels were measured via ELISA. (C) Blood was also analyzed for glucose levels. * = p ≤ 0.05 and **** = p ≤ 0.0001.


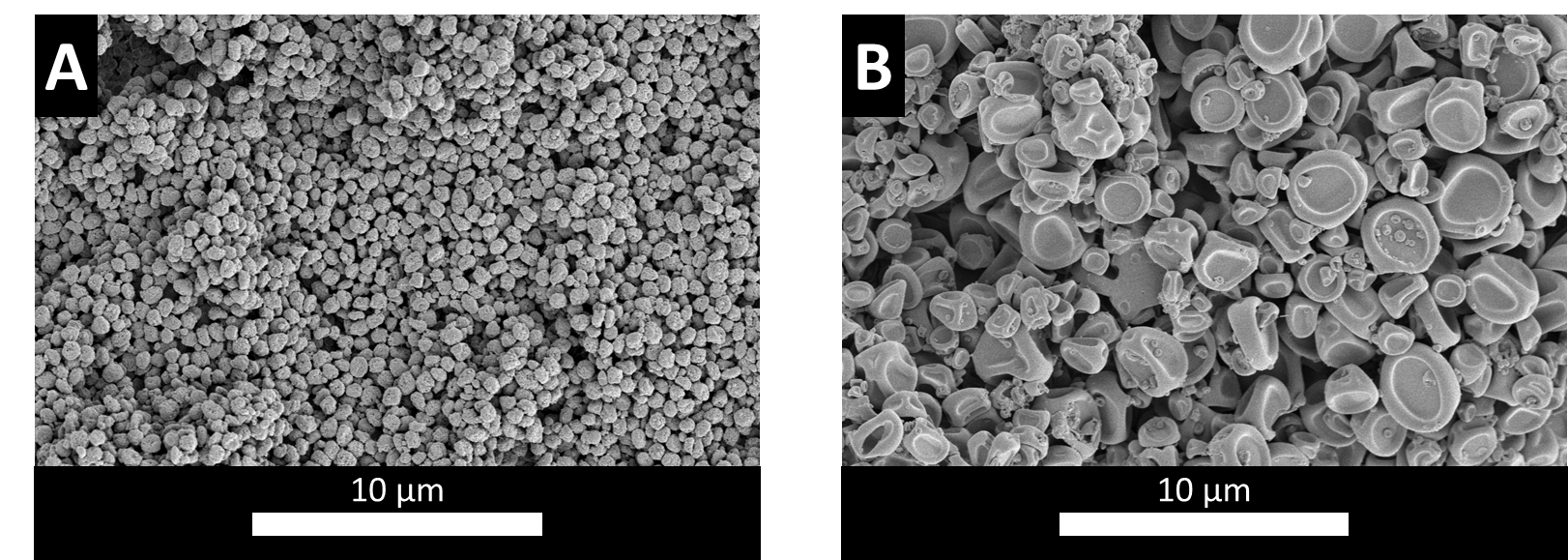


**Figure S3.** SEM micrographs of (A) cGAMP and (B) J4 loaded Ace-DEX MPs fabricated via electrospray.

|  | **P-Value** | | | | | |
| --- | --- | --- | --- | --- | --- | --- |
|  | **IgG Titer** | **TX/12 HAI Titer** | **IFN-γ (ng/mL)** | **IL-2 (ng/mL)** | **% Central Memory of CD4+** | **% Effector Memory of CD4+** |
| PBS (non-DIO) vs. PBS (DIO) | >0.9999 | >0.9999 | >0.9999 | >0.9999 | >0.9999 | 0.9984 |
| PBS (non-DIO) vs. Soluble J4 (non-DIO) | >0.9999 | >0.9999 | >0.9999 | >0.9999 | >0.9999 | >0.9999 |
| PBS (non-DIO) vs. Soluble J4 + Addavax (non-DIO) | <0.0001 | <0.0001 | >0.9999 | >0.9999 | 0.6647 | 0.4125 |
| PBS (non-DIO) vs. Soluble J4 + cGAMP MPs (non-DIO) | <0.0001 | 0.0016 | >0.9999 | 0.9519 | 0.6945 | 0.2894 |
| PBS (non-DIO) vs. J4 MPs + cGAMP MPs (non-DIO) | <0.0001 | 0.0005 | >0.9999 | 0.0017 | 0.9971 | 0.8765 |
| PBS (DIO) vs. Soluble J4 (DIO) | 0.1884 | >0.9999 | >0.9999 | >0.9999 | 0.9955 | 0.98 |
| PBS (DIO) vs. Soluble J4 + Addavax (DIO) | <0.0001 | 0.0009 | >0.9999 | >0.9999 | 0.0569 | 0.1997 |
| PBS (DIO) vs. Soluble J4 + cGAMP MPs (DIO) | <0.0001 | 0.163 | 0.985 | 0.198 | 0.5719 | 0.1601 |
| PBS (DIO) vs. J4 MPs + cGAMP MPs (DIO) | <0.0001 | 0.0316 | <0.0001 | <0.0001 | 0.6808 | 0.9659 |
| Soluble J4 (non-DIO) vs. Soluble J4 (DIO) | 0.2219 | >0.9999 | >0.9999 | >0.9999 | 0.9986 | 0.6626 |
| Soluble J4 (non-DIO) vs. Soluble J4 + Addavax (non-DIO) | <0.0001 | <0.0001 | >0.9999 | >0.9999 | 0.3292 | 0.5261 |
| Soluble J4 (non-DIO) vs. Soluble J4 + cGAMP MPs (non-DIO) | <0.0001 | 0.0045 | >0.9999 | 0.9847 | 0.3351 | 0.381 |
| Soluble J4 (non-DIO) vs. J4 MPs + cGAMP MPs (non-DIO) | <0.0001 | 0.0016 | >0.9999 | 0.0036 | 0.9385 | 0.9489 |
| Soluble J4 (DIO) vs. Soluble J4 + Addavax (DIO) | <0.0001 | 0.0009 | >0.9999 | >0.9999 | 0.0009 | 0.6868 |
| Soluble J4 (DIO) vs. Soluble J4 + cGAMP MPs (DIO) | <0.0001 | 0.163 | 0.9877 | 0.2075 | 0.0465 | 0.6029 |
| Soluble J4 (DIO) vs. J4 MPs + cGAMP MPs (DIO) | <0.0001 | 0.0316 | <0.0001 | <0.0001 | 0.0735 | >0.9999 |
| Soluble J4 + Addavax (non-DIO) vs. Soluble J4 + Addavax (DIO) | 0.0869 | 0.6422 | >0.9999 | >0.9999 | 0.9149 | 0.9041 |
| Soluble J4 + Addavax (non-DIO) vs. Soluble J4 + cGAMP MPs (non-DIO) | 0.194 | 0.5263 | >0.9999 | 0.9979 | >0.9999 | >0.9999 |
| Soluble J4 + Addavax (non-DIO) vs. J4 MPs + cGAMP MPs (non-DIO) | 0.4442 | 0.752 | >0.9999 | 0.0085 | 0.9857 | 0.9984 |
| Soluble J4 + Addavax (DIO) vs. Soluble J4 + cGAMP MPs (DIO) | 0.0187 | 0.6422 | 0.9896 | 0.2439 | 0.8961 | >0.9999 |
| Soluble J4 + Addavax (DIO) vs. J4 MPs + cGAMP MPs (DIO) | 0.8556 | 0.9617 | <0.0001 | <0.0001 | 0.8086 | 0.7542 |
| Soluble J4 + cGAMP MPs (non-DIO) vs. Soluble J4 + cGAMP MPs (DIO) | 0.4998 | 0.752 | 0.9997 | 0.8427 | >0.9999 | 0.8593 |
| Soluble J4 + cGAMP MPs (non-DIO) vs. J4 MPs + cGAMP MPs (non-DIO) | 0.0001 | >0.9999 | >0.9999 | 0.0951 | 0.9925 | 0.9943 |
| Soluble J4 + cGAMP MPs (DIO) vs. J4 MPs + cGAMP MPs (DIO) | <0.0001 | 0.9993 | <0.0001 | 0.0001 | >0.9999 | 0.6746 |
| J4 MPs + cGAMP MPs (non-DIO) vs. J4 MPs + cGAMP MPs (DIO) | 0.353 | 0.9162 | <0.0001 | 0.0187 | 0.9926 | 0.9998 |

**Supplementary Table 1.** P-values from figure 1 generated by running an ANOVA followed by Tukey’s pairwise comparisons.

**
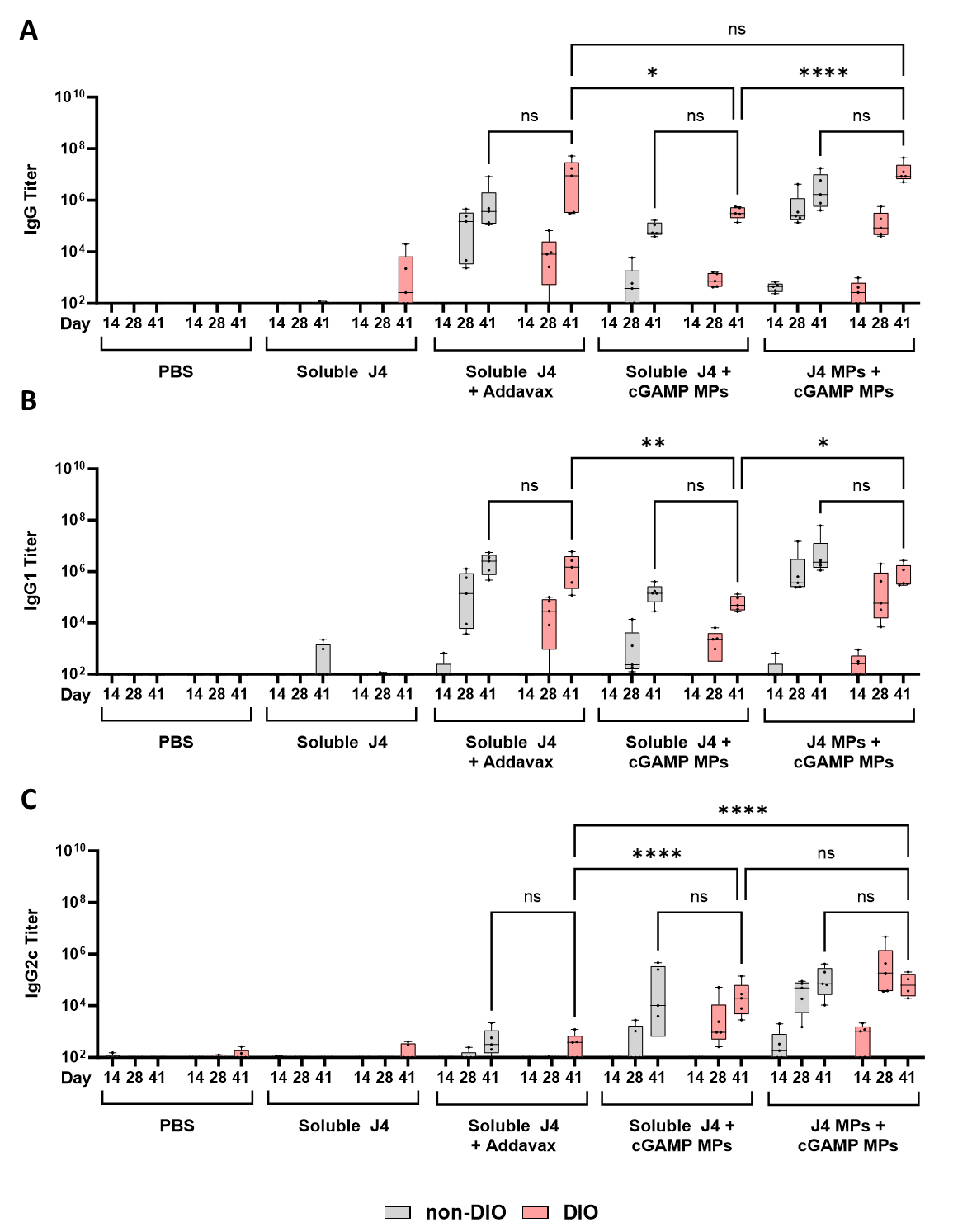
**

**Figure S4.** Mice (n = 5; non-DIO or DIO C57BL/6J) were vaccinated on a prime + boost + boost schedule (days 0, 21, and 35) with the indicated groups at doses of 1 μg cGAMP and 1 μg J4 per mouse. On days 14, 28, and 41, sera were collected and analyzed for J4-specific IgG (A), IgG1 (B) and IgG2c (C) titers via ELISA. Data is represented as mean ± SD. ns = p > 0.05, * = p ≤ 0.05, ** = p ≤ 0.01, and **** = p ≤ 0.0001. NS means not significant.

**
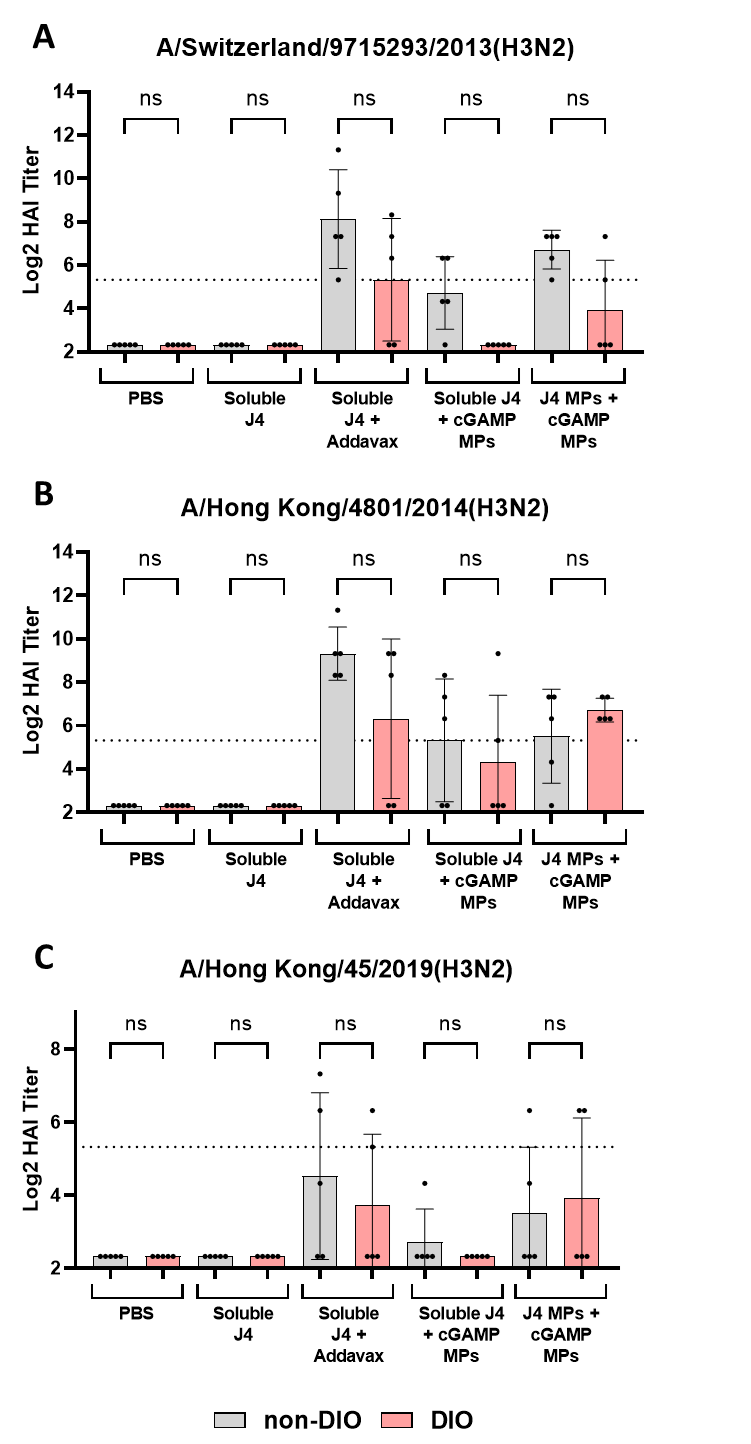
**

**Figure S5.** Mice (n = 5; non-DIO or DIO C57BL/6J) were vaccinated on a prime + boost + boost schedule (days 0, 21, and 35) with the indicated groups at doses of 1 μg cGAMP and 1 μg J4 per mouse. (B) On day 41 sera was used to determine HAI activity against A/Switzerland/9715293/2013(H3N2), A/Hong Kong/4801/2014(H3N2), and A/Hong Kong/45/2019(H3N2). (A-C) The dotted line represents the seroconversion titer value of 1:40. Data is represented as mean ± SD. ns = p > 0.05. NS means not significant.

**
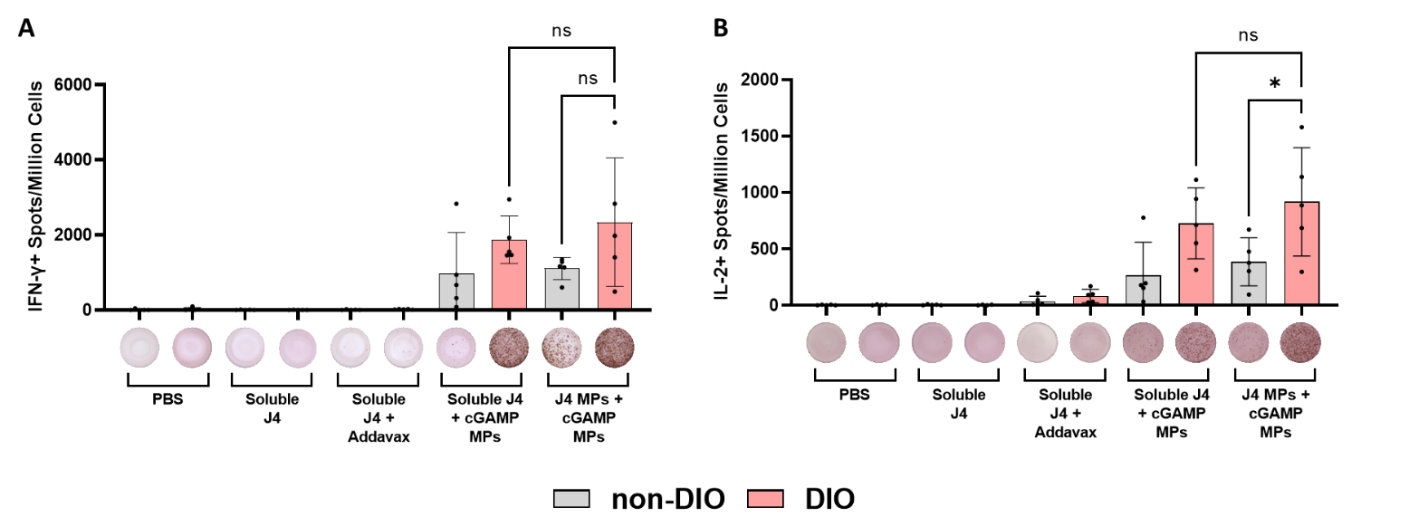
**

**Figure S6.** Mice (n = 5; non-DIO or DIO C57BL/6J) were vaccinated on a prime + boost + boost schedule (days 0, 21, and 35) with the indicated groups at doses of 1 μg cGAMP and 1 μg J4 per mouse. On day 42, mice were humanely euthanized to collect spleens. Splenocytes were stimulated with J4 for 36 h after which the antigen specific production of (A) IFN-γ and (B) IL-2 was measured via ELISpot. Data is represented as mean ± SD. ns = p > 0.05 and * = p ≤ 0.05. NS means not significant.


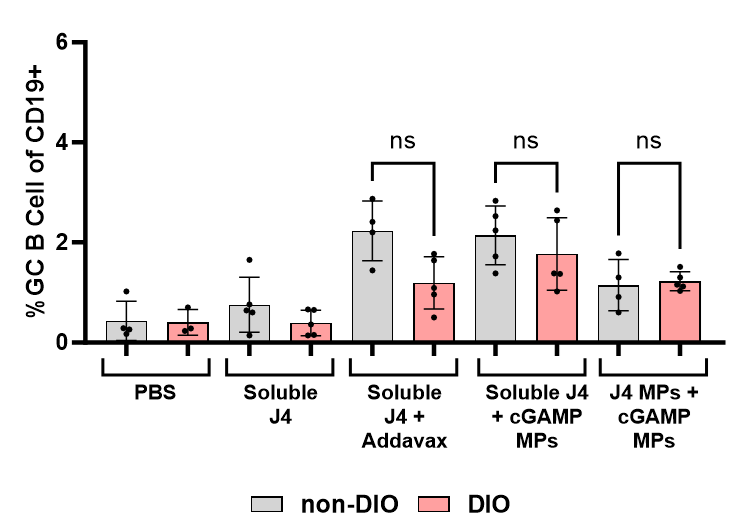


**Figure S7.** Mice (n = 5; non-DIO or DIO C57BL/6J) were vaccinated on a prime + boost + boost schedule (days 0, 21, and 35) with the indicated groups at doses of 1 μg cGAMP and 1 μg J4 per mouse. Lymph nodes were harvested on day 42 to measure the expansion germinal center (GC) B cells (CD10+, GL7+, and CD38-) via flow cytometry. Data is represented as mean ± SD. ns = p > 0.05. NS means not significant.

|  | **P-Value** | | | | | |
| --- | --- | --- | --- | --- | --- | --- |
|  | **IgG Titer** | **TX/12 HAI Titer** | **IFN-γ (ng/mL)** | **IL-2 (pg/mL)** | **% Central Memory of CD4+** | **% Effector Memory of CD4+** |
| PBS (non-Aged) vs. PBS (Aged) | >0.9999 | >0.9999 | >0.9999 | >0.9999 | 0.0339 | <0.0001 |
| PBS (non-Aged) vs. Soluble J4 (non-Aged) | 0.7137 | 0.9985 | >0.9999 | >0.9999 | 0.8375 | >0.9999 |
| PBS (non-Aged) vs. Soluble J4 + Addavax (non-Aged) | <0.0001 | <0.0001 | >0.9999 | >0.9999 | 0.0483 | >0.9999 |
| PBS (non-Aged) vs. Soluble J4 + cGAMP MPs (non-Aged) | <0.0001 | <0.0001 | >0.9999 | >0.9999 | 0.1423 | >0.9999 |
| PBS (non-Aged) vs. J4 MPs + cGAMP MPs (non-Aged) | <0.0001 | <0.0001 | <0.0001 | <0.0001 | 0.3437 | 0.9858 |
| PBS (Aged) vs. Soluble J4 (Aged) | >0.9999 | >0.9999 | >0.9999 | 0.9994 | >0.9999 | >0.9999 |
| PBS (Aged) vs. Soluble J4 + Addavax (Aged) | 0.1488 | 0.9932 | >0.9999 | >0.9999 | 0.9958 | 0.9994 |
| PBS (Aged) vs. Soluble J4 + cGAMP MPs (Aged) | 0.5275 | 0.9932 | >0.9999 | 0.9992 | 0.9979 | 0.6911 |
| PBS (Aged) vs. J4 MPs + cGAMP MPs (Aged) | <0.0001 | 0.0189 | >0.9999 | 0.6322 | >0.9999 | 0.5505 |
| Soluble J4 (non-Aged) vs. Soluble J4 (Aged) | 0.6598 | 0.9978 | >0.9999 | 0.9711 | 0.2019 | <0.0001 |
| Soluble J4 (non-Aged) vs. Soluble J4 + Addavax (non-Aged) | <0.0001 | <0.0001 | >0.9999 | >0.9999 | 0.7711 | >0.9999 |
| Soluble J4 (non-Aged) vs. Soluble J4 + cGAMP MPs (non-Aged) | <0.0001 | <0.0001 | >0.9999 | >0.9999 | 0.9552 | 0.9995 |
| Soluble J4 (non-Aged) vs. J4 MPs + cGAMP MPs (non-Aged) | <0.0001 | <0.0001 | <0.0001 | <0.0001 | 0.9982 | 0.9502 |
| Soluble J4 (Aged) vs. Soluble J4 + Addavax (Aged) | 0.1488 | 0.9932 | >0.9999 | >0.9999 | 0.8595 | 0.9995 |
| Soluble J4 (Aged) vs. Soluble J4 + cGAMP MPs (Aged) | 0.5275 | 0.9932 | >0.9999 | 0.9099 | >0.9999 | 0.6053 |
| Soluble J4 (Aged) vs. J4 MPs + cGAMP MPs (Aged) | <0.0001 | 0.0189 | >0.9999 | 0.2016 | >0.9999 | 0.4469 |
| Soluble J4 + Addavax (non-Aged) vs. Soluble J4 + Addavax (Aged) | <0.0001 | <0.0001 | >0.9999 | >0.9999 | 0.9998 | <0.0001 |
| Soluble J4 + Addavax (non-Aged) vs. Soluble J4 + cGAMP MPs (non-Aged) | 0.4081 | 0.9716 | >0.9999 | >0.9999 | >0.9999 | >0.9999 |
| Soluble J4 + Addavax (non-Aged) vs. J4 MPs + cGAMP MPs (non-Aged) | 0.9997 | 0.4945 | <0.0001 | <0.0001 | 0.9946 | 0.9963 |
| Soluble J4 + Addavax (Aged) vs. Soluble J4 + cGAMP MPs (Aged) | 0.9994 | >0.9999 | >0.9999 | 0.9953 | 0.6277 | 0.9489 |
| Soluble J4 + Addavax (Aged) vs. J4 MPs + cGAMP MPs (Aged) | 0.0265 | 0.155 | >0.9999 | 0.5015 | 0.9325 | 0.8686 |
| Soluble J4 + cGAMP MPs (non-Aged) vs. Soluble J4 + cGAMP MPs (Aged) | <0.0001 | <0.0001 | >0.9999 | >0.9999 | 0.7591 | <0.0001 |
| Soluble J4 + cGAMP MPs (non-Aged) vs. J4 MPs + cGAMP MPs (non-Aged) | 0.1048 | 0.9921 | <0.0001 | <0.0001 | >0.9999 | 0.9998 |
| Soluble J4 + cGAMP MPs (Aged) vs. J4 MPs + cGAMP MPs (Aged) | 0.0023 | 0.155 | >0.9999 | 0.9678 | 0.9998 | >0.9999 |
| J4 MPs + cGAMP MPs (non-Aged) vs. J4 MPs + cGAMP MPs (Aged) | <0.0001 | 0.0496 | <0.0001 | <0.0001 | 0.7972 | <0.0001 |

**Supplementary Table 2.** P-values from figure 3 generated by running an ANOVA followed by Tukey’s pairwise comparisons.


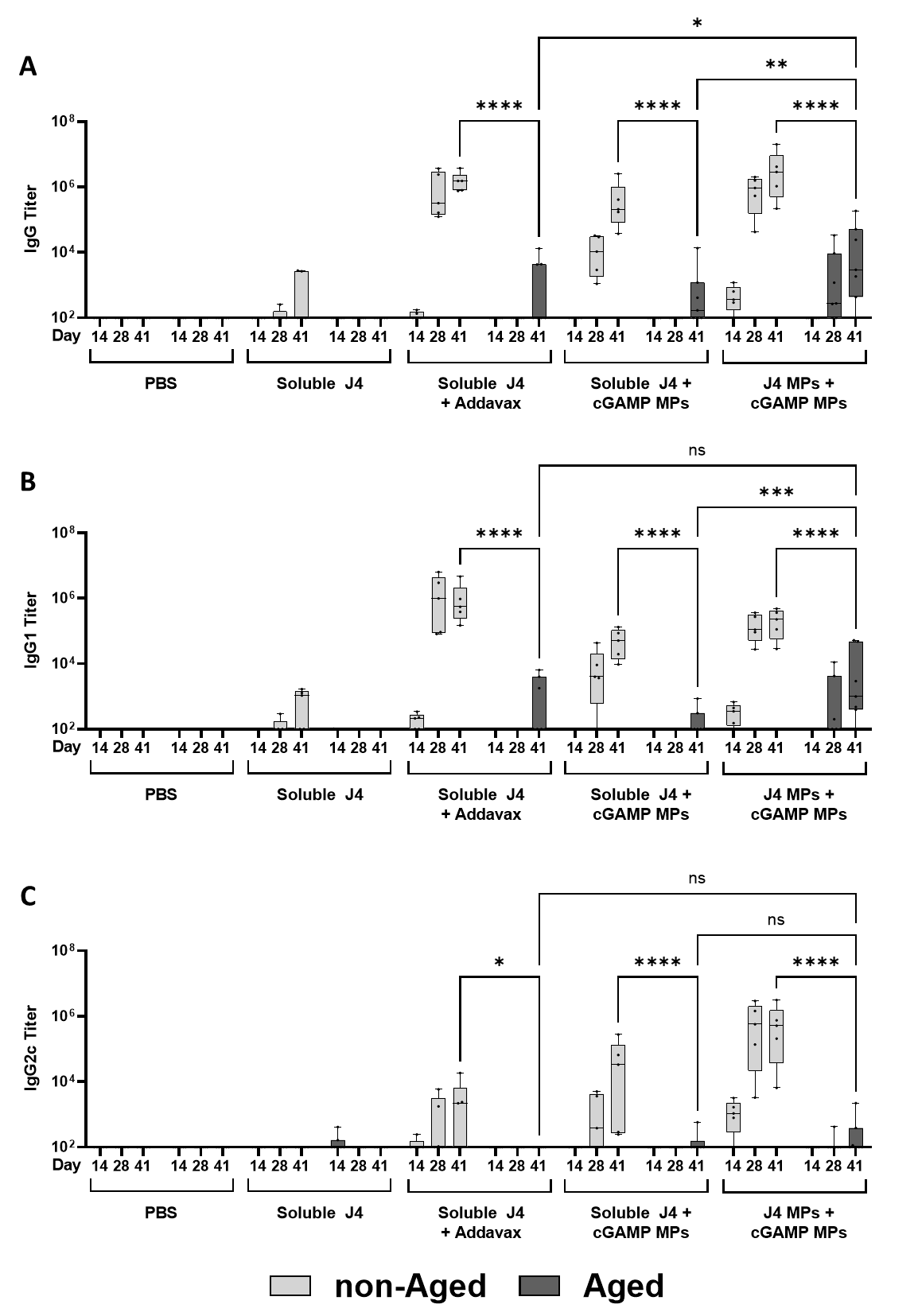


**Figure S8.** Mice (n = 5 non-aged or n = 7 aged C57BL/6J) were vaccinated on a prime + boost + boost schedule (days 0, 21, and 35) with the indicated groups at doses of 1 μg cGAMP and 1 μg J4 per mouse. On days 14, 28, and 41, sera were collected and analyzed for J4-specific IgG (A), IgG1 (B) and IgG2c (C) titers via ELISA. Data is represented as mean ± SD. ns = p > 0.05, * = p ≤ 0.05, ** = p ≤ 0.01, and **** = p ≤ 0.0001. NS means not significant.

**
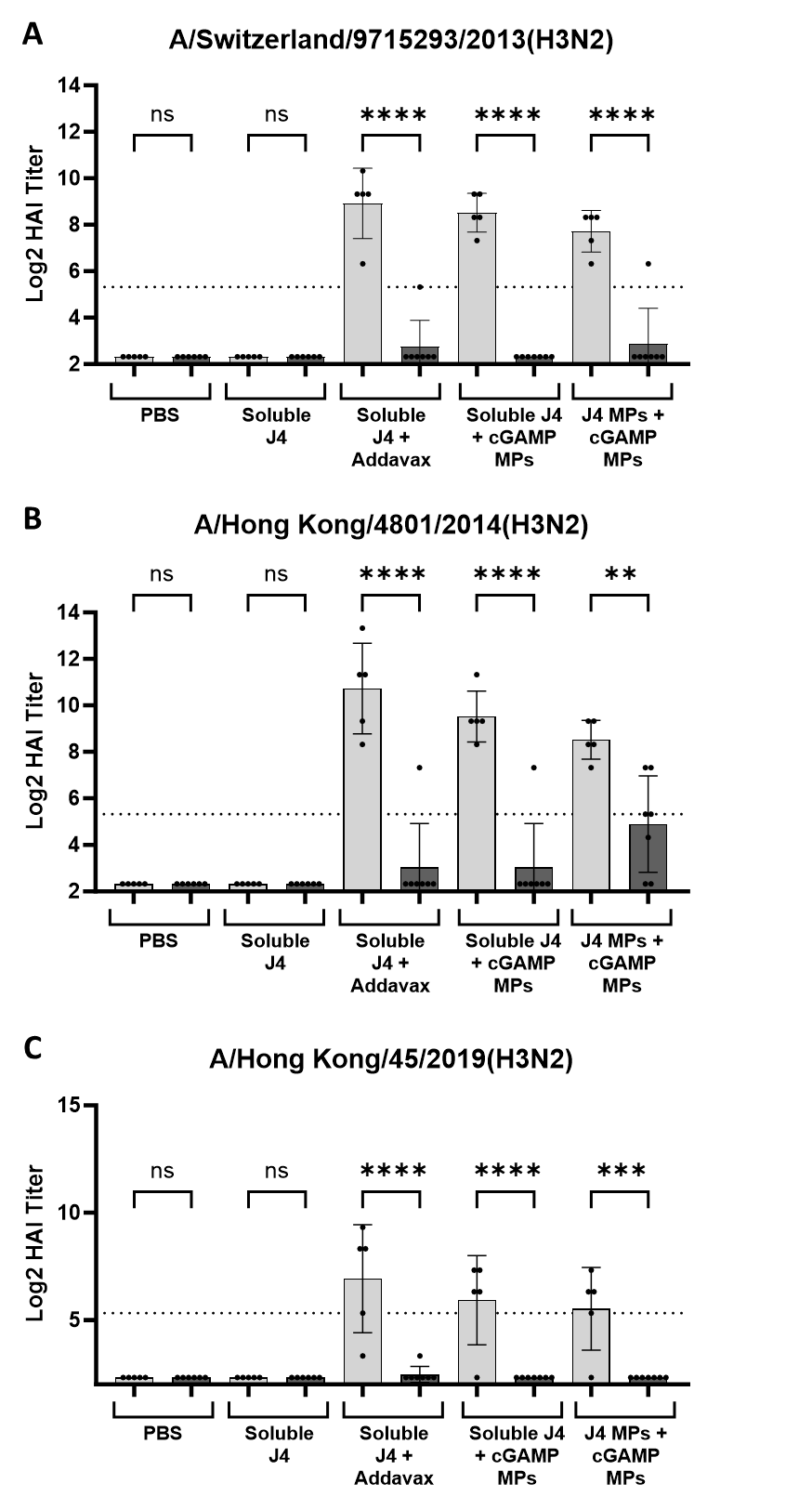
**

**Figure S9.** Mice (n = 5 non-aged or n = 7 aged C57BL/6J) were vaccinated on a prime + boost + boost schedule (days 0, 21, and 35) with the indicated groups at doses of 1 μg cGAMP and 1 μg J4 per mouse. (B) On day 41 sera was used to determine HAI activity against A/Switzerland/9715293/2013(H3N2), A/Hong Kong/4801/2014(H3N2), and A/Hong Kong/45/2019(H3N2). (A-C) The dotted line represents the seroconversion titer value of 1:40. Data is represented as mean ± SD. ns = p > 0.05, ** = p ≤ 0.01, *** = p ≤ 0.001 and **** = p ≤ 0.0001. NS means not significant.

**
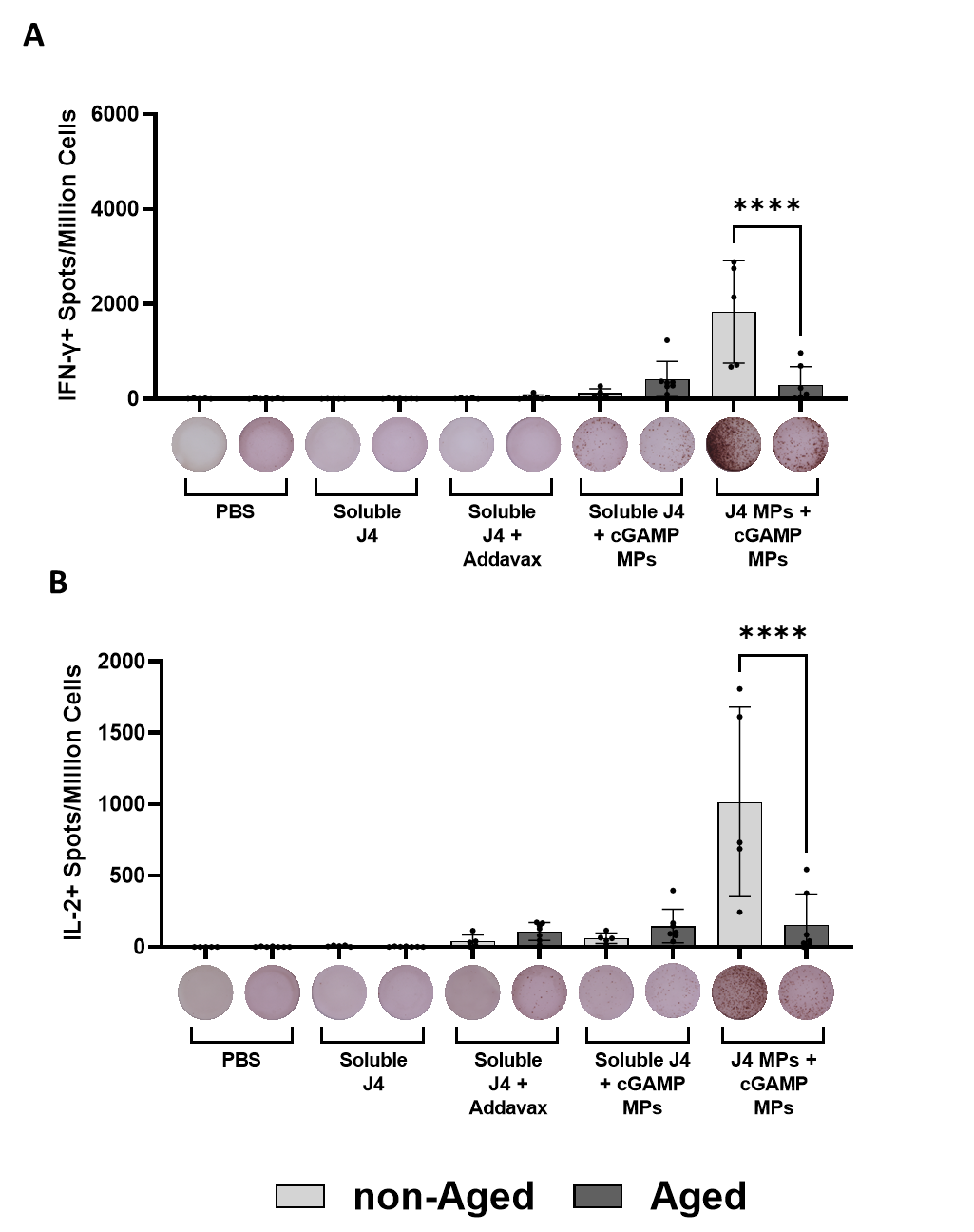
**

**Figure S10.** Mice (n = 5 non-aged or n = 7 aged C57BL/6J) were vaccinated on a prime + boost + boost schedule (days 0, 21, and 35) with the indicated groups at doses of 1 μg cGAMP and 1 μg J4 per mouse. On day 42, mice were humanely euthanized to collect spleens. Splenocytes were stimulated with J4 for 36 h after which the antigen specific production of (A) IFN-γ and (B) IL-2 was measured via ELISpot. Data is represented as mean ± SD. **** = p ≤ 0.0001.

**
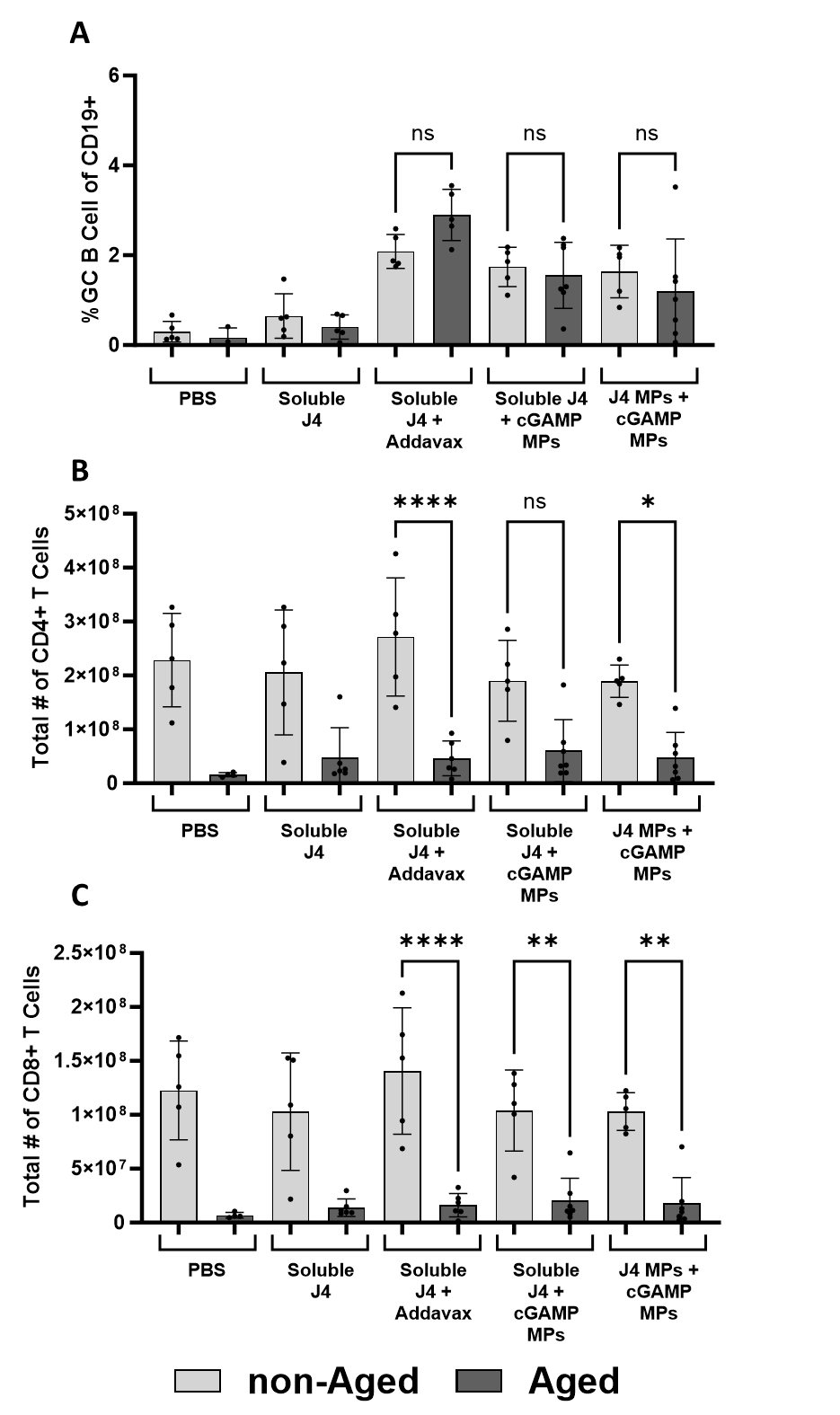
**

**Figure S11.** Mice (n = 5 non-aged or n = 7 aged C57BL/6J) were vaccinated on a prime + boost + boost schedule (days 0, 21, and 35) with the indicated groups at doses of 1 μg cGAMP and 1 μg J4 per mouse. (A) Lymph nodes were harvested on day 42 to measure the expansion germinal center (GC) B cells (CD10+, GL7+, and CD38-) as well as the total number of CD4+ and CD8+ T cells via flow cytometry. Data is represented as mean ± SD. ns = p > 0.05, * = p ≤ 0.05 ** = p ≤ 0.01, and **** = p ≤ 0.0001. NS means not significant.

|  | **P-Value** | | | | | |
| --- | --- | --- | --- | --- | --- | --- |
|  | **IgG Titer** | **TX/12 HAI Titer** | **IFN-γ (ng/mL)** | **IL-2 (pg/mL)** | **% Central Memory of CD4+** | **% Effector Memory of CD4+** |
| PBS (non-CP) vs. PBS (CP) | >0.9999 | >0.9999 | >0.9999 | 0.9967 | 0.9997 | 0.9535 |
| PBS (non-CP) vs. Soluble J4 (non-CP) | 0.084 | 0.9823 | >0.9999 | 0.996 | 0.5127 | >0.9999 |
| PBS (non-CP) vs. Soluble J4 + Addavax (non-CP) | <0.0001 | <0.0001 | >0.9999 | >0.9999 | 0.2607 | 0.0542 |
| PBS (non-CP) vs. Soluble J4 + cGAMP MPs (non-CP) | <0.0001 | <0.0001 | >0.9999 | >0.9999 | 0.0678 | 0.0422 |
| PBS (non-CP) vs. J4 MPs + cGAMP MPs (non-CP) | <0.0001 | <0.0001 | 0.001 | 0.0042 | 0.0182 | <0.0001 |
| PBS (CP) vs. Soluble J4 (CP) | >0.9999 | >0.9999 | >0.9999 | >0.9999 | 0.9989 | >0.9999 |
| PBS (CP) vs. Soluble J4 + Addavax (CP) | <0.0001 | 0.3461 | >0.9999 | >0.9999 | 0.9875 | 0.0173 |
| PBS (CP) vs. Soluble J4 + cGAMP MPs (CP) | 0.0026 | >0.9999 | 0.9801 | 0.9928 | >0.9999 | 0.8207 |
| PBS (CP) vs. J4 MPs + cGAMP MPs (CP) | <0.0001 | 0.9823 | 0.4211 | 0.0235 | 0.9878 | 0.0113 |
| Soluble J4 (non-CP) vs. Soluble J4 (CP) | 0.084 | 0.9823 | >0.9999 | >0.9999 | 0.5822 | 0.9305 |
| Soluble J4 (non-CP) vs. Soluble J4 + Addavax (non-CP) | <0.0001 | <0.0001 | >0.9999 | 0.9998 | >0.9999 | 0.07 |
| Soluble J4 (non-CP) vs. Soluble J4 + cGAMP MPs (non-CP) | <0.0001 | 0.0001 | >0.9999 | >0.9999 | 0.9833 | 0.055 |
| Soluble J4 (non-CP) vs. J4 MPs + cGAMP MPs (non-CP) | <0.0001 | <0.0001 | 0.0009 | 0.0001 | 0.8359 | <0.0001 |
| Soluble J4 (CP) vs. Soluble J4 + Addavax (CP) | <0.0001 | 0.3461 | >0.9999 | >0.9999 | 0.7609 | 0.0283 |
| Soluble J4 (CP) vs. Soluble J4 + cGAMP MPs (CP) | 0.0026 | >0.9999 | 0.9802 | 0.9963 | >0.9999 | 0.9061 |
| Soluble J4 (CP) vs. J4 MPs + cGAMP MPs (CP) | <0.0001 | 0.9823 | 0.4214 | 0.0302 | 0.7382 | 0.0193 |
| Soluble J4 + Addavax (non-CP) vs. Soluble J4 + Addavax (CP) | <0.0001 | <0.0001 | >0.9999 | >0.9999 | 0.0073 | 0.7075 |
| Soluble J4 + Addavax (non-CP) vs. Soluble J4 + cGAMP MPs (non-CP) | 0.9914 | 0.0188 | >0.9999 | >0.9999 | 0.9997 | >0.9999 |
| Soluble J4 + Addavax (non-CP) vs. J4 MPs + cGAMP MPs (non-CP) | 0.9998 | 0.0962 | 0.0011 | 0.0015 | 0.9731 | 0.0074 |
| Soluble J4 + Addavax (CP) vs. Soluble J4 + cGAMP MPs (CP) | 0.9439 | 0.3461 | 0.9816 | 0.996 | 0.8565 | 0.4605 |
| Soluble J4 + Addavax (CP) vs. J4 MPs + cGAMP MPs (CP) | 0.9918 | 0.9441 | 0.4292 | 0.0293 | >0.9999 | >0.9999 |
| Soluble J4 + cGAMP MPs (non-CP) vs. Soluble J4 + cGAMP MPs (CP) | <0.0001 | <0.0001 | 0.993 | 0.9999 | 0.0547 | >0.9999 |
| Soluble J4 + cGAMP MPs (non-CP) vs. J4 MPs + cGAMP MPs (non-CP) | 0.832 | 0.9996 | 0.0018 | 0.0007 | >0.9999 | 0.0099 |
| Soluble J4 + cGAMP MPs (CP) vs. J4 MPs + cGAMP MPs (CP) | 0.3761 | 0.9823 | 0.9796 | 0.2681 | 0.8438 | 0.421 |
| J4 MPs + cGAMP MPs (non-CP) vs. J4 MPs + cGAMP MPs (CP) | <0.0001 | <0.0001 | 0.4779 | 0.9186 | 0.0001 | 0.4775 |

**Supplementary Table 3.** P-values from figure 5 generated by running an ANOVA followed by Tukey’s pairwise comparisons.

**
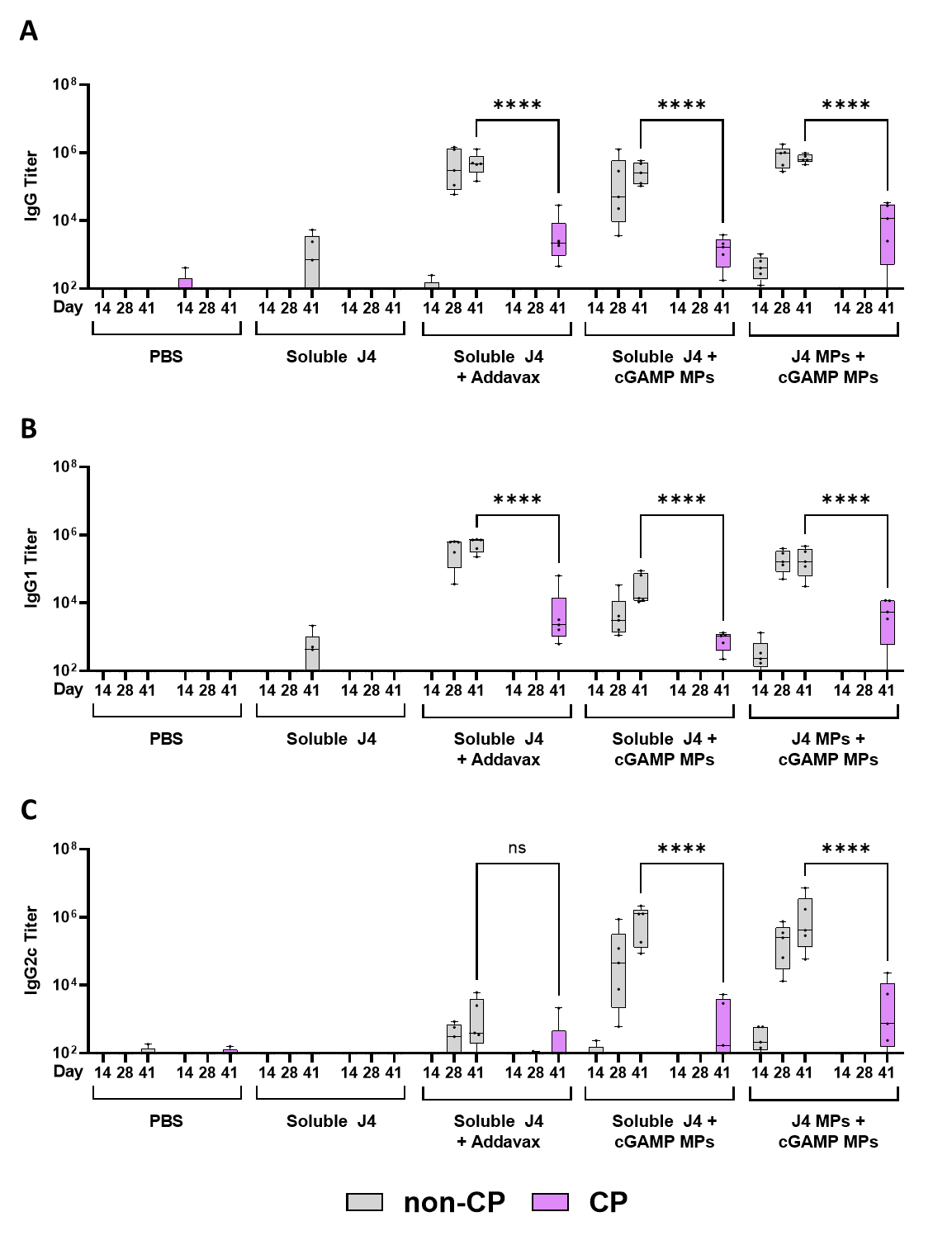
**

**Figure S12.** Mice (n = 5; non-cyclophosphamide treated (non-CP) or cyclophosphamide treated (CP) C57BL/6J) were vaccinated on a prime + boost + boost schedule (days 0, 21, and 35) with the indicated groups at doses of 1 μg cGAMP and 1 μg J4 per mouse. On days 14, 28, and 41, sera were collected and analyzed for J4-specific IgG (A), IgG1 (B) and IgG2c (C) titers via ELISA. Data is represented as mean ± SD. ns = p > 0.05 and **** = p ≤ 0.0001. NS means not significant.

**
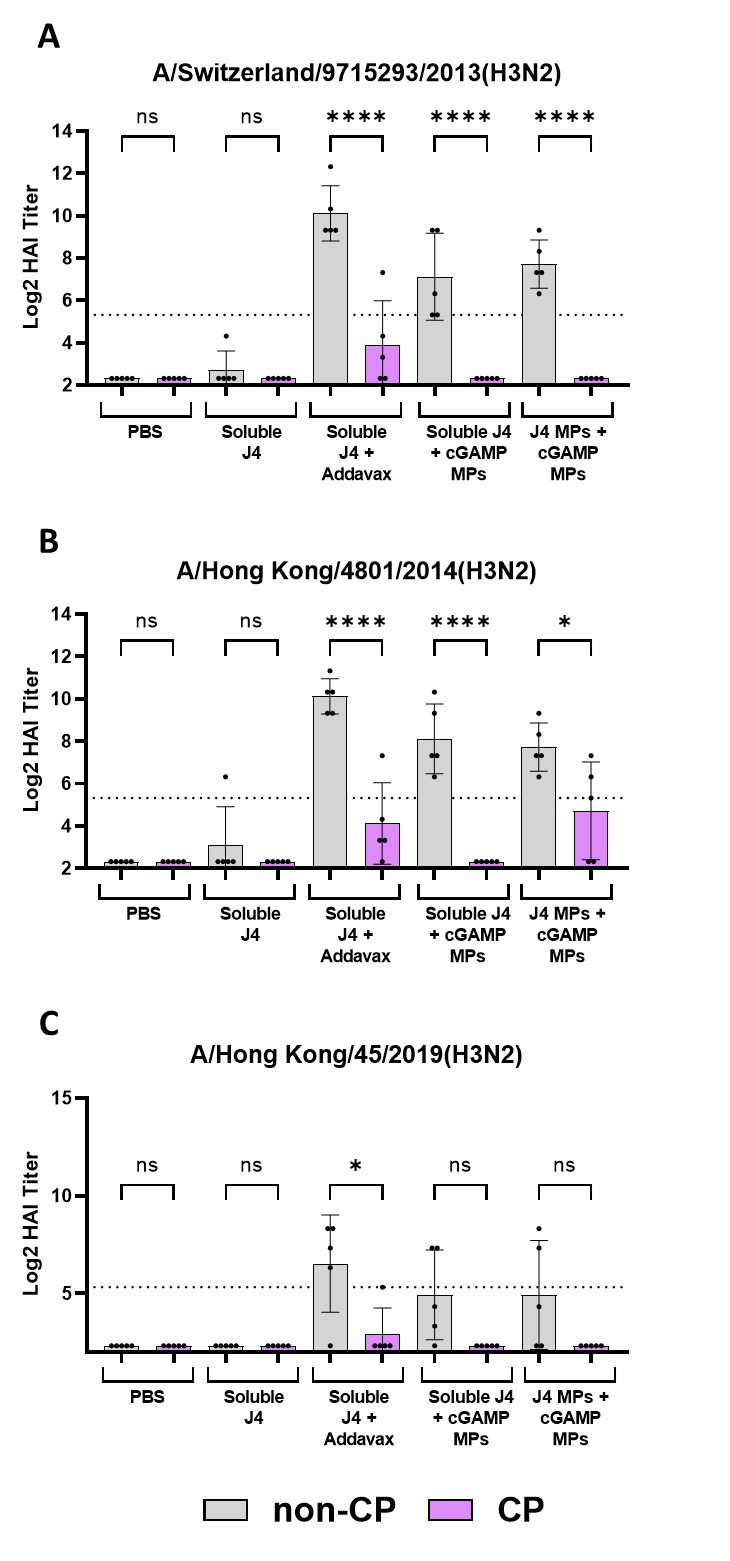
**

**Figure S13.** Mice (n = 5; non-cyclophosphamide treated (non-CP) or cyclophosphamide treated (CP) C57BL/6J) were vaccinated on a prime + boost + boost schedule (days 0, 21, and 35) with the indicated groups at doses of 1 μg cGAMP and 1 μg J4 per mouse. (B) On day 41 sera was used to determine HAI activity against A/Switzerland/9715293/2013(H3N2), A/Hong Kong/4801/2014(H3N2), and A/Hong Kong/45/2019(H3N2). (A-C) The dotted line represents the seroconversion titer value of 1:40. Data is represented as mean ± SD. ns = p > 0.05, * = p ≤ 0.05, and **** = p ≤ 0.0001. NS means not significant.

**
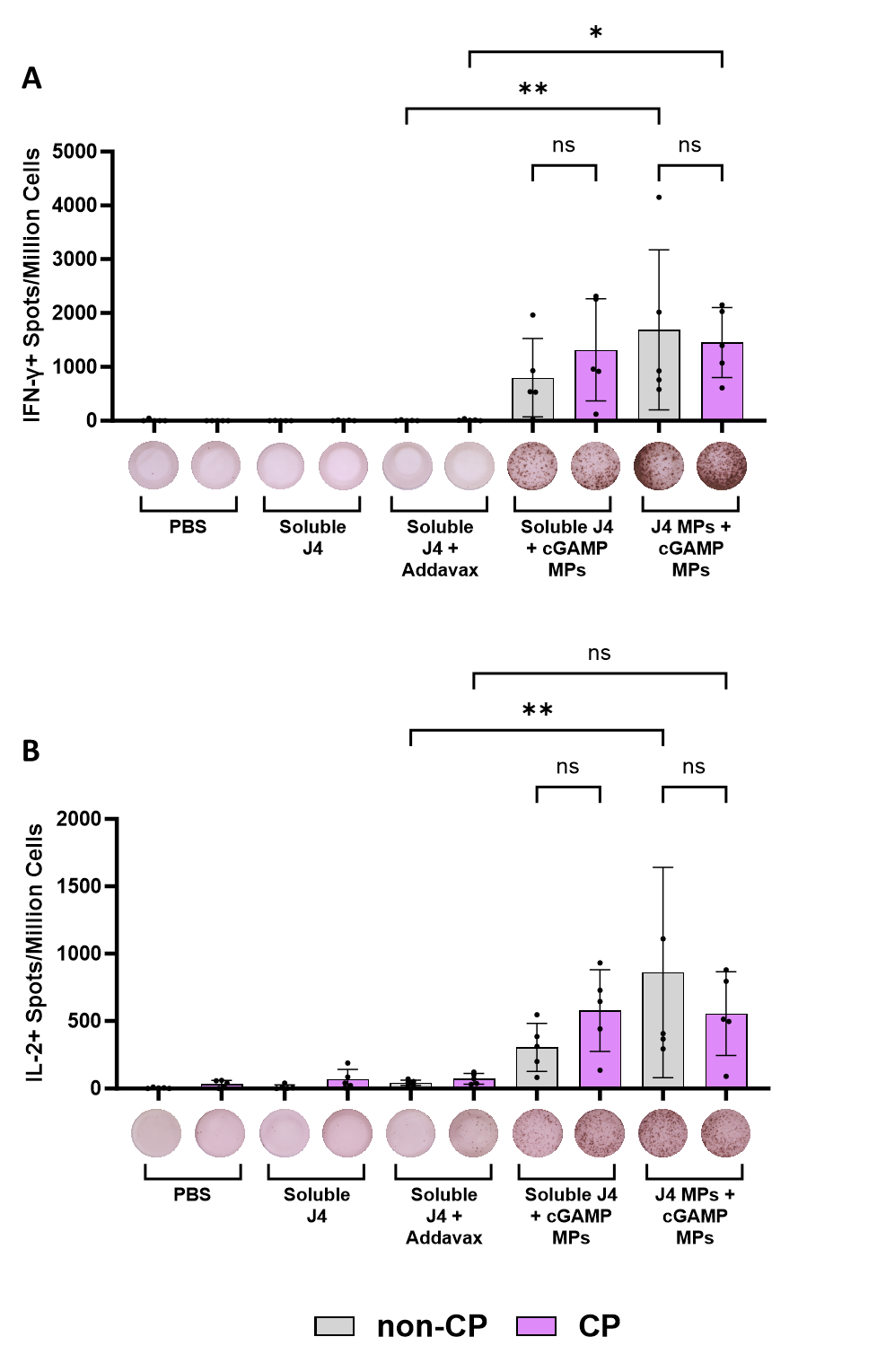
**

**Figure S14.** Mice (n = 5; non-cyclophosphamide treated (non-CP) or cyclophosphamide treated (CP) C57BL/6J) were vaccinated on a prime + boost + boost schedule (days 0, 21, and 35) with the indicated groups at doses of 1 μg cGAMP and 1 μg J4 per mouse. On day 42, mice were humanely euthanized to collect spleens. Splenocytes were stimulated with J4 for 36 h after which the antigen specific production of (A) IFN-γ and (B) IL-2 was measured via ELISpot. Data is represented as mean ± SD. ns = p > 0.05, * = p ≤ 0.05, and ** = p ≤ 0.01. NS means not significant.

**
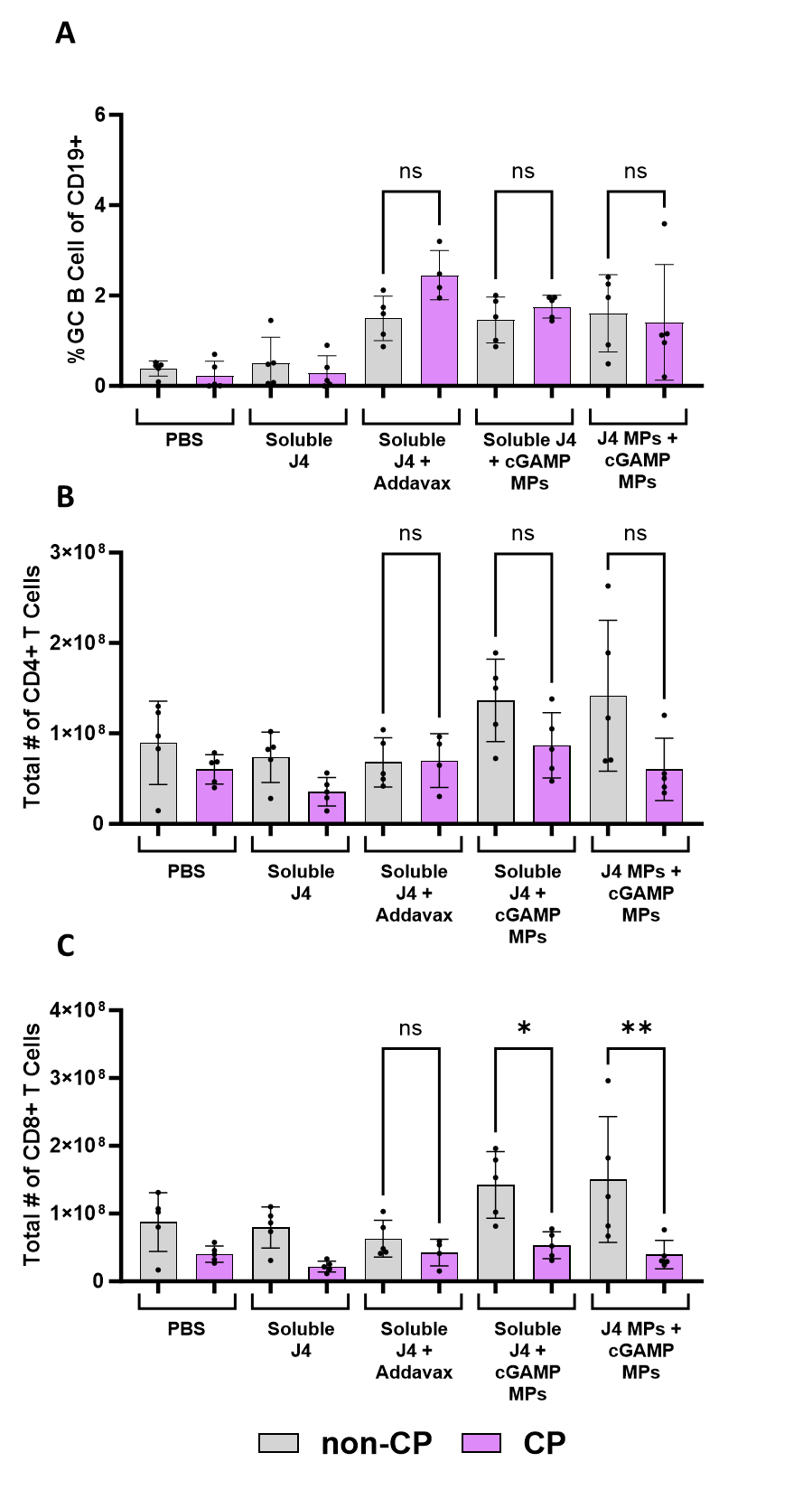
**

**Figure S15.** Mice (n = 5; non-cyclophosphamide treated (non-CP) or cyclophosphamide treated (CP) C57BL/6J) were vaccinated on a prime + boost + boost schedule (days 0, 21, and 35) with the indicated groups at doses of 1 μg cGAMP and 1 μg J4 per mouse. (A) Lymph nodes were harvested on day 42 to measure the expansion germinal center (GC) B cells (CD10+, GL7+, and CD38-) as well as the total number of CD4+ and CD8+ T cells via flow cytometry. Data is represented as mean ± SD. ns = p > 0.05, * = p ≤ 0.05, and ** = p ≤ 0.01. NS means not significant.

|  | **P-Value** | | | |
| --- | --- | --- | --- | --- |
|  | **IgG Titer** | **TX/12 HAI Titer** | **IFN-γ (ng/mL)** | **IL-2 (pg/mL)** |
| **CC012** | <0.0001 | 0.9104 | >0.9999 | >0.9999 |
| **CC013** | 0.9665 | >0.9999 | >0.9999 | >0.9999 |
| **CC027** | 0.1652 | 0.3182 | >0.9999 | 0.998 |
| **CC029** | >0.9999 | >0.9999 | 0.0135 | 0.0637 |
| **CC032** | 0.9127 | >0.9999 | 0.0508 | 0.1178 |
| **CC035** | >0.9999 | 0.8839 | 0.9982 | 0.3666 |
| **CC060** | 0.9996 | 0.9998 | 0.9957 | 0.9714 |
| **CC061** | >0.9999 | 0.6203 | 0.4909 | 0.4387 |
| **CC071** | 0.249 | 0.9998 | 0.7597 | >0.9999 |
| **CC072** | 0.9575 | >0.9999 | >0.9999 | >0.9999 |
| **CC078** | 0.5637 | 0.9962 | 0.9997 | >0.9999 |
| **CC084** | 0.3839 | >0.9999 | >0.9999 | 0.9986 |

**Supplementary Table 4.** P-values from figure 6 generated by running an ANOVA followed by Tukey’s pairwise comparisons to compare vaccination with either soluble J4 + Addavax or soluble J4 + cGAMP MPs within the 12 CC strains examined.

**
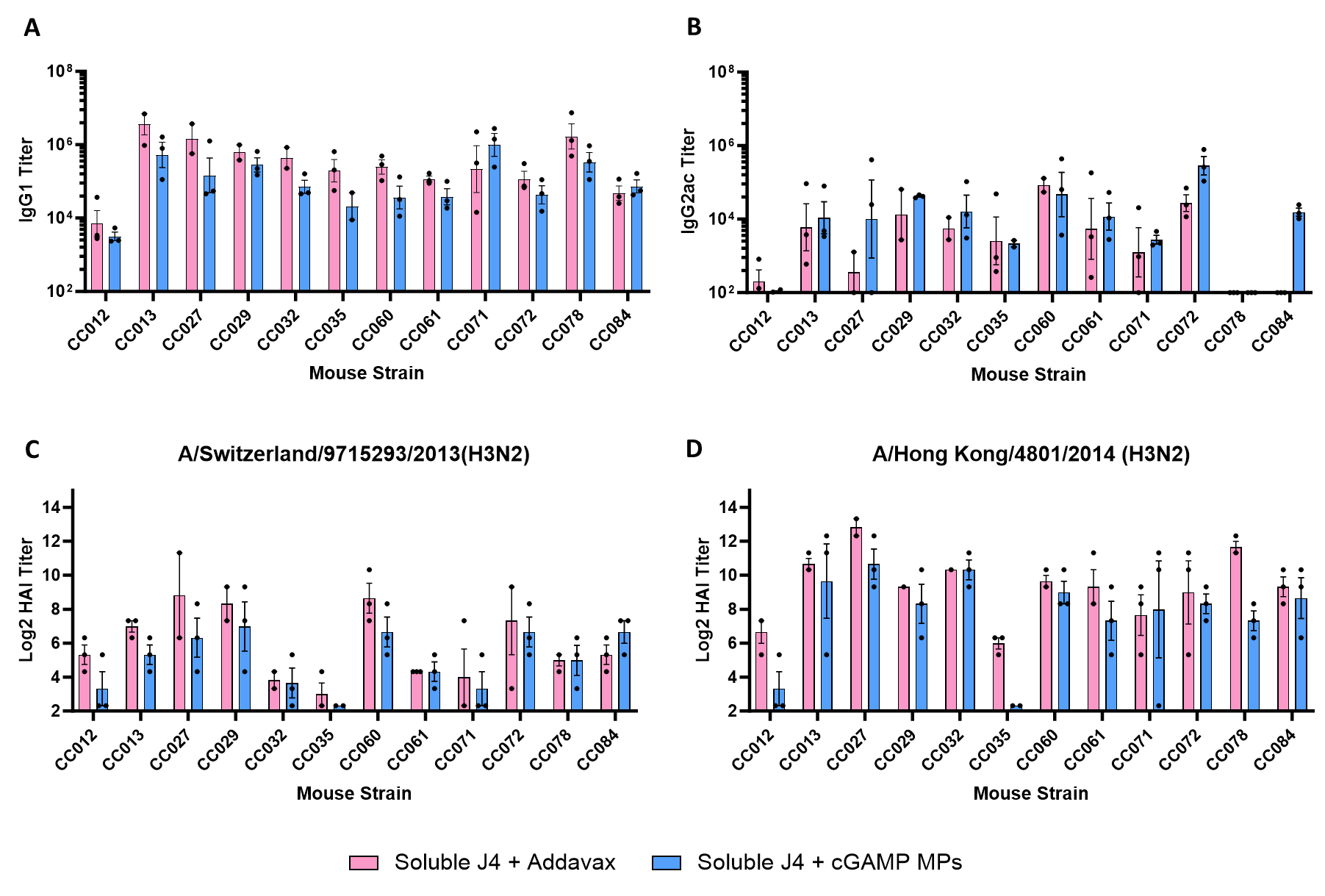
**

**Figure S16.** Mice (n = 3; 12 different collaborative cross strains) were vaccinated on a prime + boost + boost schedule (days 0, 21, and 35) with the indicated groups at doses of 1 μg cGAMP and 1 μg J4 per mouse. On day 41, sera were collected and analyzed for J4-specific (A) IgG1 and (B) IgG2ac titers via ELISA. (B) Day 41 sera was also used to determine HAI activity against A/Switzerland/9715293/2013(H3N2). Data is represented as mean ± SD.

**
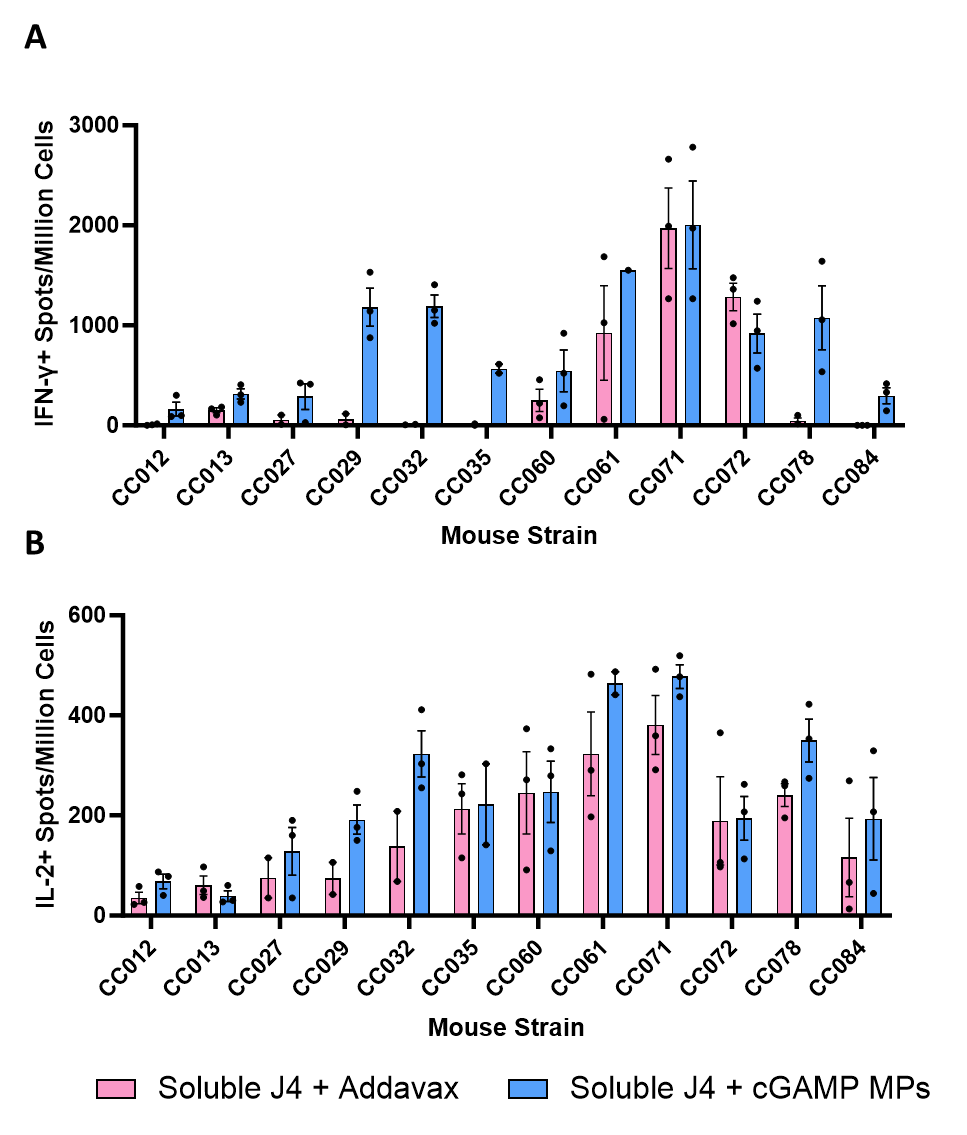
**

**Figure S17.** Mice (n = 3; 12 different collaborative cross strains) were vaccinated on a prime + boost + boost schedule (days 0, 21, and 35) with the indicated groups at doses of 1 μg cGAMP and 1 μg J4 per mouse. On day 42, mice were humanely euthanized to collect spleens. Splenocytes were stimulated with J4 for 36 h after which the antigen specific production of (A) IFN-γ and (B) IL-2 was measured via ELISpot. Data is represented as mean ± SD.

| **Phenotype** | **Heritability for Soluble J4 + Addavax** | **Heritability for Soluble J4 + cGAMP MPs** |
| --- | --- | --- |
| Anti-J4 IgG Titer | 87.7% | 80.8% |
| Anti-J4 IgG1 Titer | 72.9% | 79.4% |
| Anti-J4 IgG2ac Titer | 70.1% | 76.9% |
| IFN-γ+ Spots/Million Cells | 89.1% | 79.6% |
| IL-2+ Spots/Million Cells | 65.8% | 80.3% |

**Supplementary Table 5.** Summary of broad-sense heritability for IgG titer, IgG1 titer, IgG2ac titer, IFN-γ+ spots/million cells, and IL-2+ spots/million cells based on whether the group was vaccinated with soluble J4 + Addavax or soluble J4 + cGAMP MPs.
